# Ultrasensitive chemiluminescent neuraminidase probe for rapid screening and identification of small-molecules with antiviral activity against influenza A virus in mammalian cells†

**DOI:** 10.1039/d2sc03460c

**Published:** 2022-09-26

**Authors:** Omri Shelef, Sara Gutkin, Daniel Feder, Ariel Ben-Bassat, Michal Mandelboim, Yoni Haitin, Nir Ben-Tal, Eran Bacharach, Doron Shabat

**Affiliations:** School of Chemistry, Raymond and Beverly Sackler Faculty of Exact Sciences, Tel-Aviv University Tel Aviv 69978 Israel chdoron@tauex.tau.ac.il +972 (0)3 640 9293 +972 (0)3 640 8340; School of Neurobiology, Biochemistry and Biophysics, George S. Wise Faculty of Life Sciences, Tel Aviv University Tel Aviv 69978 Israel; The Shmunis School of Biomedicine and Cancer Research, George S. Wise Faculty of Life Sciences, Tel Aviv University Tel Aviv 69978 Israel; Department of Physiology and Pharmacology, Sackler Faculty of Medicine, Tel Aviv University Tel Aviv Israel; Central Virology Laboratory, Sheba Medical Center Tel Hashomer Ramat-Gan 52620 Israel; School of Public Health, Sackler Faculty of Medicine, Tel Aviv University Tel Aviv 69978 Israel

## Abstract

Influenza A virus is the most virulent influenza subtype and is associated with large-scale global pandemics characterized by high levels of morbidity and mortality. Developing simple and sensitive molecular methods for detecting influenza viruses is critical. Neuraminidase, an exo-glycosidase displayed on the surface of influenza virions, is responsible for the release of the virions and their spread in the infected host. Here, we present a new phenoxy-dioxetane chemiluminescent probe (CLNA) that can directly detect neuraminidase activity. The probe exhibits an effective turn-on response upon reaction with neuraminidase and produces a strong emission signal at 515 nm with an extremely high signal-to-noise ratio. Comparison measurements of our new probe with previously reported analogous neuraminidase optical probes showed superior detection capability in terms of response time and sensitivity. Thus, as far as we know, our probe is the most sensitive neuraminidase probe known to date. The chemiluminescence turn-on response produced by our neuraminidase probe enables rapid screening for small molecules that inhibit viral replication through different mechanisms as validated directly in influenza A-infected mammalian cells using the known inhibitors oseltamivir and amantadine. We expect that our new chemiluminescent neuraminidase probe will prove useful for various applications requiring neuraminidase detection including drug discovery assays against various influenza virus strains in mammalian cells.

## Abstract 2

A new chemiluminescence neuraminidase probe enables rapid screening of small molecules that inhibit viral replication, directly in influenza A-infected mammalian cells.

## Graphical abstract

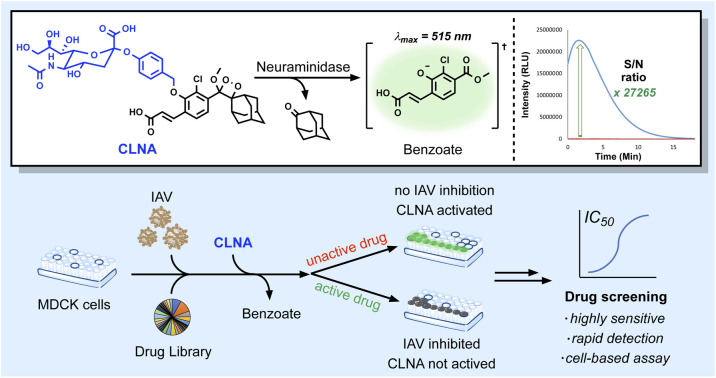

## Introduction

Influenza viruses cause respiratory infections in humans and are responsible for up to 500 000 worldwide deaths annually.^1^ In the last century, influenza viruses posed severe threats to public health, causing the 1918 and 2009 (H1N1), 1957 (H2N2), and 1968 (H3N2) pandemics.^2^ Each of these pandemics was caused by the influenza A virus (IAV), considered the most virulent influenza subtype. This subtype is associated with seasonal epidemics and more persistent transmission than subtypes B, C, and D, and occasional pandemics characterized by high levels of morbidity and mortality.^3^ Standard quantification methods for influenza viruses include hemagglutination assays,^4^ cytopathic effect (CPE) assays in infected cells,^5^ detection of viral antigens,^6^ and amplification of the viral RNA (by RT-PCR).^7^ Viral antigen detection and RT-PCR are precise and reliable; nevertheless, due to viral genetic reassortment, frequent adaptation of the antibodies and the primer sequences are required during a pandemic.^3,8^ Furthermore, considering the time-consuming procedure and the requirement for skilled personnel, developing new rapid and simple detection methods is highly important.^9,10^

**Fig. 1:**
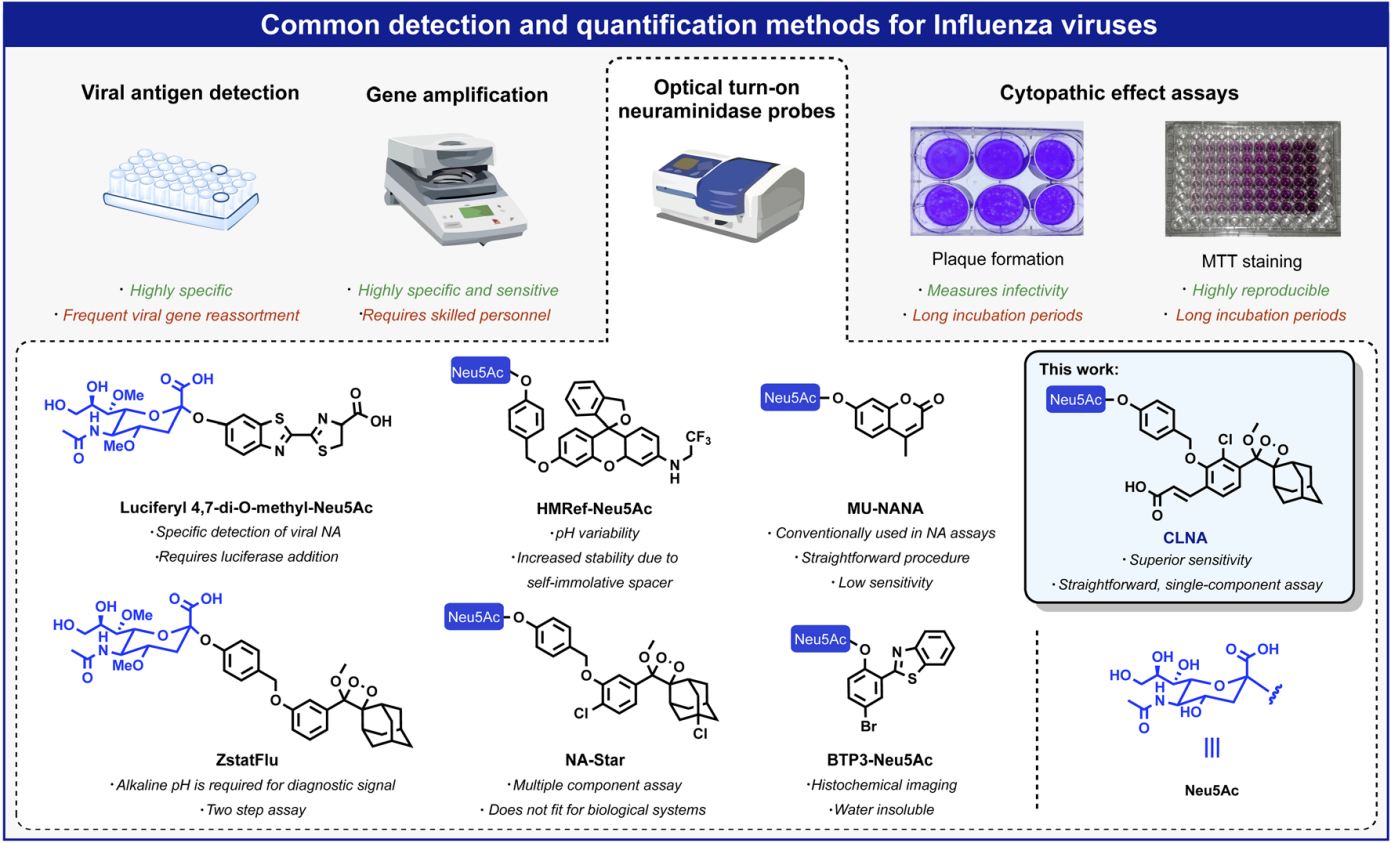
Overview of commonly used methods for detecting and quantifying influenza viruses: viral antigen detection, gene amplification, CPE assays, and optical probes based on fluorescent and chemiluminescent dyes.

The IAV virion displays on its surface three main envelope proteins: hemagglutinin (HA), neuraminidase (NA), and matrix 2 (M2). NA is an exo-glycosidase that is responsible for the hydrolytic removal of sialic acid residues from glycoconjugates. The removal of sialic acids by NA from sialylated cellular receptors and newly synthesized HA and NA prevents the aggregation of nascent virion progenies on the producer cells and thus, contributes to the efficient release of the IAV particles and their spread in the infected host.^3^ One common approach for detecting viral replication is based on optical probes that detect the catalytic activity of neuraminidase. Such probes are composed of an optical dye masked by the neuraminidase substrate *N*-acetylneuraminic acid (Neu5Ac). Enzymatic removal of *N*-acetylneuraminic acid by neuraminidase results in the release of the free dye and a turn-on optical response. Several such turn-on probes have been reported in the literature with optical signals produced by fluorescence, bioluminescence, or chemiluminescence (Fig. 1).^11^ Probes HMRef-Neu5Ac, MU-NANA, and BTP3-Neu5Ac are based on xanthene, coumarin, and 2-(benzothiazole-2-yl)-4-bromophenyl fluorescence dyes, respectively;^12–15^ the bioluminescence probe, Luciferyl4,7-di-O-methyl-Neu5Ac, is activated through light-emission produced as a result of a reaction between free luciferin and luciferase in the presence of ATP, while probes NA-Star and ZstatFlu are based on the 1,2-dioxetane chemiluminescent luminophore.^16–18^

Chemiluminescent assays are among the most sensitive methods currently employed to detect enzymatic activity. Since an excitation light source is unnecessary, autofluorescence and light scattering do not occur, and high sensitivity can be achieved due to exceptionally high signal-to-noise ratios.^19^ Probes NA-Star and ZstatFlu are composed of a masked form of the Schaap adamantyl-1,2-dioxetane luminophore and produce a chemiluminescence turn-on signal upon reaction with neuraminidase.^20^ However, the excited intermediate, formed during the chemiexcitation of the Schaap adamantyl-1,2-dioxetane luminophore, is quenched by water molecules. Thus, an assay based on this probe requires the presence of an amphiphilic micellar component and a fluorescent dye amplifier. About five years ago, our group discovered that incorporation of an acrylate substituent at the *ortho* position of a phenoxy-adamantyl-1,2-dioxetane prevents water-mediated quenching of the excited intermediate and amplifies the light-emission intensity of the chemiluminescent luminophore by up to 3000-fold.^21–27^ Importantly, this groundbreaking development enabled the use of chemiluminescent probes as a single component with no required additives.^28–34^ Numerous research groups worldwide, including ours, took advantage of the *ortho*-substituted phenoxy-adamantyl-1,2-dioxetane luminophore to develop useful chemiluminescent probes for use in cells and *in vivo* assays.^35–43^ Here we report a new ultrasensitive chemiluminescent neuraminidase probe, CLNA, and its use for rapid screening and identification of small molecules with antiviral activity against IAV in mammalian cells.

## Results and discussion

**Fig. 2:**
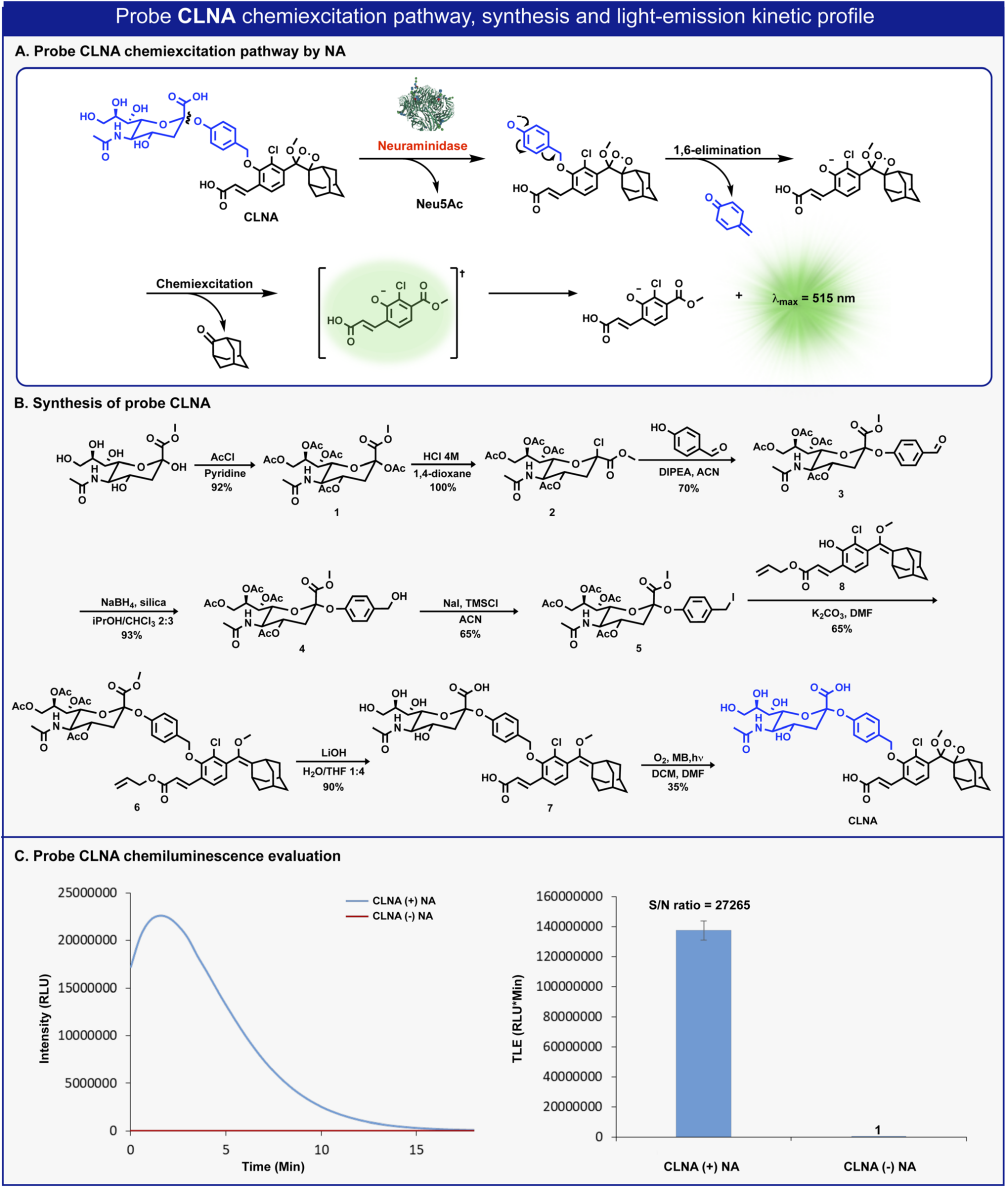
(A) Chemiexcitation disassembly pathway of CLNA upon reaction with neuraminidase. (B) Chemical synthesis of probe CLNA. (C) Chemiluminescence kinetic profile (left) and total light emitted (right) obtained by CLNA [10 μM] in 10% DMSO in PBS pH 7.4 at 27 °C with and without *C. perfringens* neuraminidase [0.1 U mL^−1^].

The molecular structure of CLNA and its chemiluminescence activation pathway are shown in Fig. 2A. The probe is composed of the NA substrate, Neu5Ac, conjugated through a short self-immolative linker to a 1,2-adamantylidene-dioxetane luminophore bearing an acrylic acid substituent. Upon the enzymatic hydrolysis of the glycosidic bond by NA, the linker undergoes spontaneous 1,6-elimination to release a phenolate intermediate. The latter undergoes a chemiexcitation process to form an excited benzoate species, which decays to its ground-state through the emission of a green photon.

The synthesis of CLNA was achieved as described in Fig. 2B. The five hydroxyl groups of Neu5Ac-methyl ester were protected with acetyl-chloride to afford penta-acetate-ester 1. The anomeric acetate of 1 was then substituted with chlorine by treatment with hydrochloric acid to yield chloride 2. Nucleophilic substitution of chloride 2 with 4-hydroxybenzaldehyde afforded aldehyde 3. The aldehyde was reduced using sodium borohydride, in the presence of silica, to yield benzyl-alcohol 4, which was then treated with sodium iodide and trimethylsilyl chloride to produce benzyl-iodide 5. Nucleophilic substitution of benzyl-iodide 5 with the previously synthesized phenol enolether 8 (ref. 44) afforded ether 6. The four acetyl-ester groups and the allyl acrylate of 6 were then hydrolyzed using lithium hydroxide to give compound 7, which was subsequently oxidized by singlet oxygen to yield CLNA.

Initially, we sought to evaluate the light-emission turn-on response and the chemiluminescence kinetic profile of CLNA upon reaction with neuraminidase. CLNA was incubated in an aqueous buffer (PBS, pH 7.4) in the presence or the absence of recombinant bacterial neuraminidase (from *C. perfringens*). The chemiluminescence light emission signal, measured over time, and the total emitted photons are presented in Fig. 2C. Predictably, probe CLNA presented a rapid turn-on response upon incubation of CLNA with neuraminidase, with an initial light-emission signal increasing to a maximum and a subsequent decay. No light-emission signal was observed in the control reaction without neuraminidase. Remarkably, the total light-emission signal measured for probe CLNA in the presence of neuraminidase was about 27 000-fold greater than that observed in the absence of the enzyme. This is an exceptionally large signal-to-noise ratio (S/N), even for a chemiluminescence probe, most likely due to the high hydrolytic stability of the molecular structure of CLNA.

**Fig. 3:**
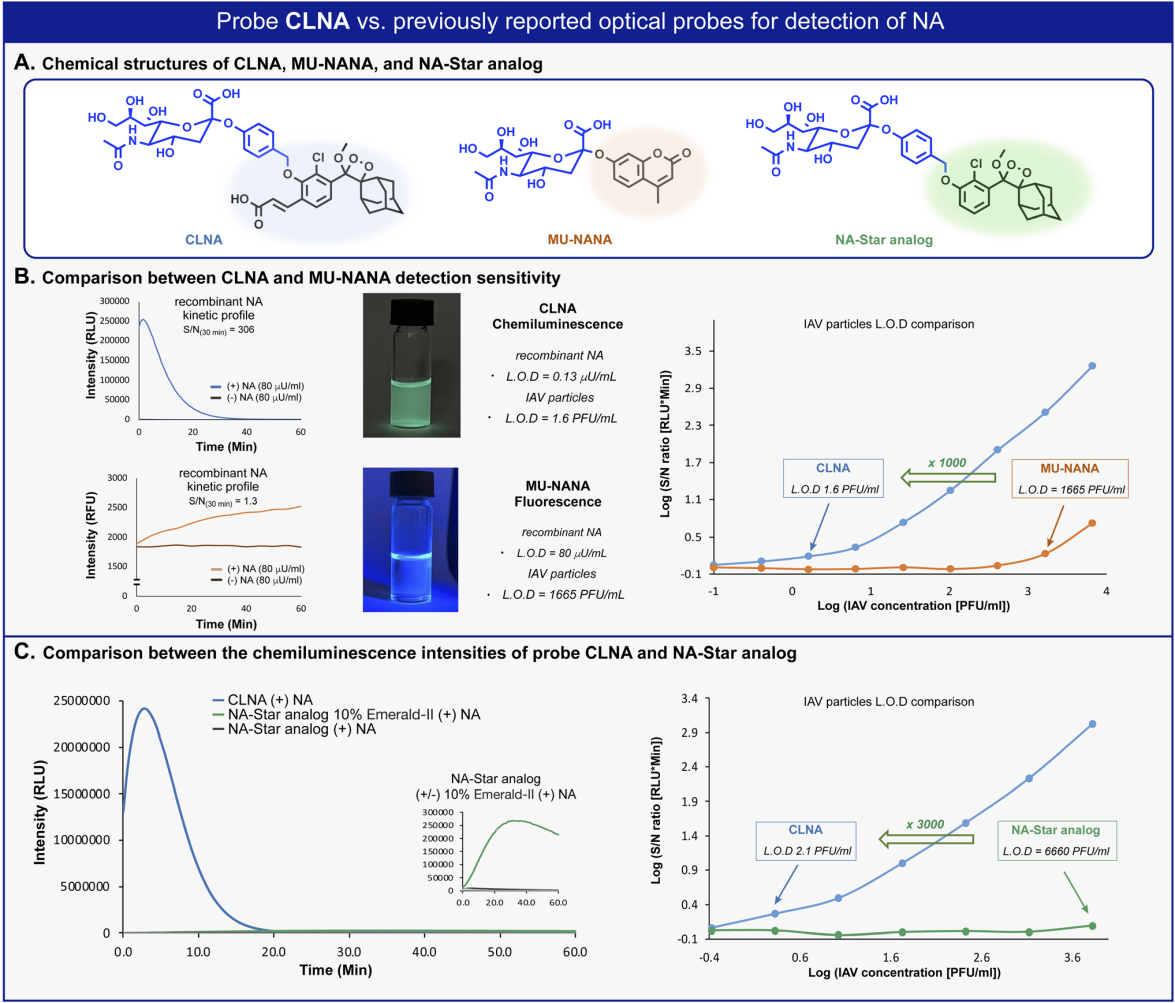
(A) Chemical structures of CLNA and commercially available fluorescent and chemiluminescent probes. (B) Left: Kinetic profile of CLNA and MU-NANA in the presence of *C. perfringens* neuraminidase [80 μU mL^−1^]. Middle: Images of CLNA chemiluminescence and MU-NANA fluorescence excited at 254 nm. Right: S/N at 30 min for CLNA [10 μM] and MU-NANA [10 μM] at IAV particle concentrations ranging from 0.1 to 104.1 PFU mL^−1^ in 1% DMSO in PBS, pH 7.4 at 37 °C. (C) Left: Kinetic profile of NA-Star analog in the presence of *C. perfringens* neuraminidase [0.1 U mL^−1^]. Right: S/N at 30 min for CLNA [10 μM] and NA-Star analog [10 μM] with 10% Emerald-II™ enhancer at IAV particle concentrations ranging from 0.1 to 104.1 PFU mL^−1^ in 1% DMSO in PBS, pH 7.4 at 37 °C.

We next sought to compare the detection sensitivity of probe CLNA for neuraminidase with that of currently existing optical assays. MU-NANA is a commonly used fluorescent neuraminidase substrate, and NA-Star analog is an equivalent of the commercially available chemiluminescence probe NA-Star. The S/N values of CLNA and the fluorogenic commercial probe MU-NANA were determined in the presence of various concentrations of recombinant neuraminidase. Expectedly, CLNA exhibited a limit of detection (LOD) value, about 615-fold greater than that of MU-NANA (Fig. 3B, left and Fig. S1–S3†). We next evaluated the sensitivity of probe CLNA for detection of IAV particles in comparison to that of MU-NANA (Fig. 3B, right, and Fig. S13, S14†). CLNA had 1000-fold higher detection sensitivity (LOD = 1.6 PFU mL^−1^) than MU-NANA (LOD = 1665 PFU mL^−1^) for the same viral supernatant. The detection sensitivity of probe CLNA was also compared to that of NA-Star analog in the presence of neuraminidase and IAV particles. Remarkably, chemiluminescence probe CLNA, exhibited S/N value of 4017, which is 47-fold higher than that of the NA-Star analog with 10% Emerald-II™ enhancer for the detection of recombinant NA (Fig. 3C, left and Fig. S16†) and 3000-fold higher detection sensitivity than NA-Star analog for the detection of IAV particles (Fig. 3C, right). These results demonstrate the superior ability of the new chemiluminescence probe CLNA to detect neuraminidase activity *versus* current fluorescence and chemiluminescence probes.

Currently, there are three main classes of FDA-approved drugs for the treatment of IAV infection: adamantane derivatives, amantadine, and rimantadine, which target the wild-type M2 ion channel; neuraminidase inhibitors such as oseltamivir, peramivir, and zanamivir; and the newly approved xofluza, which targets the endonuclease cap-snatching activity of viral RNA polymerase.^45–47^ However, because of the high mutation rate of influenza viruses, which leads to the emergence of resistant strains, there is a need for new small-molecule-based drugs for influenza treatment.^11^ The enzymatic activity of the influenza neuraminidase can be monitored in a cell-based assay to identify potential small-molecule inhibitors of influenza virus replication.^48^ Assays conducted with the commercially available chemiluminescence probes NA-Star and ZstatFlu must be performed in the presence of Emerald-II™ enhancer.^49^ This additive is composed of a fluorescence dye and a detergent polymer, which is incompatible with cell assays due to its high toxicity. In contrast, a major potential advantage of probe CLNA (beyond its extraordinary detection sensitivity for neuraminidase) is its compatibility with cell-based assays. Thus, we next sought to evaluate the accuracy and sensitivity of CLNA in screening small molecules as potential antiviral drugs for the influenza virus using IAV-infected mammalian cells. Since NA levels correlate with virus replication levels, quantifying NA enzymatic activity using CLNA should provide a direct and accurate measurement for viral replication inhibition by any tested molecule, regardless of the inhibitory mechanism.^48^

**Fig. 4:**
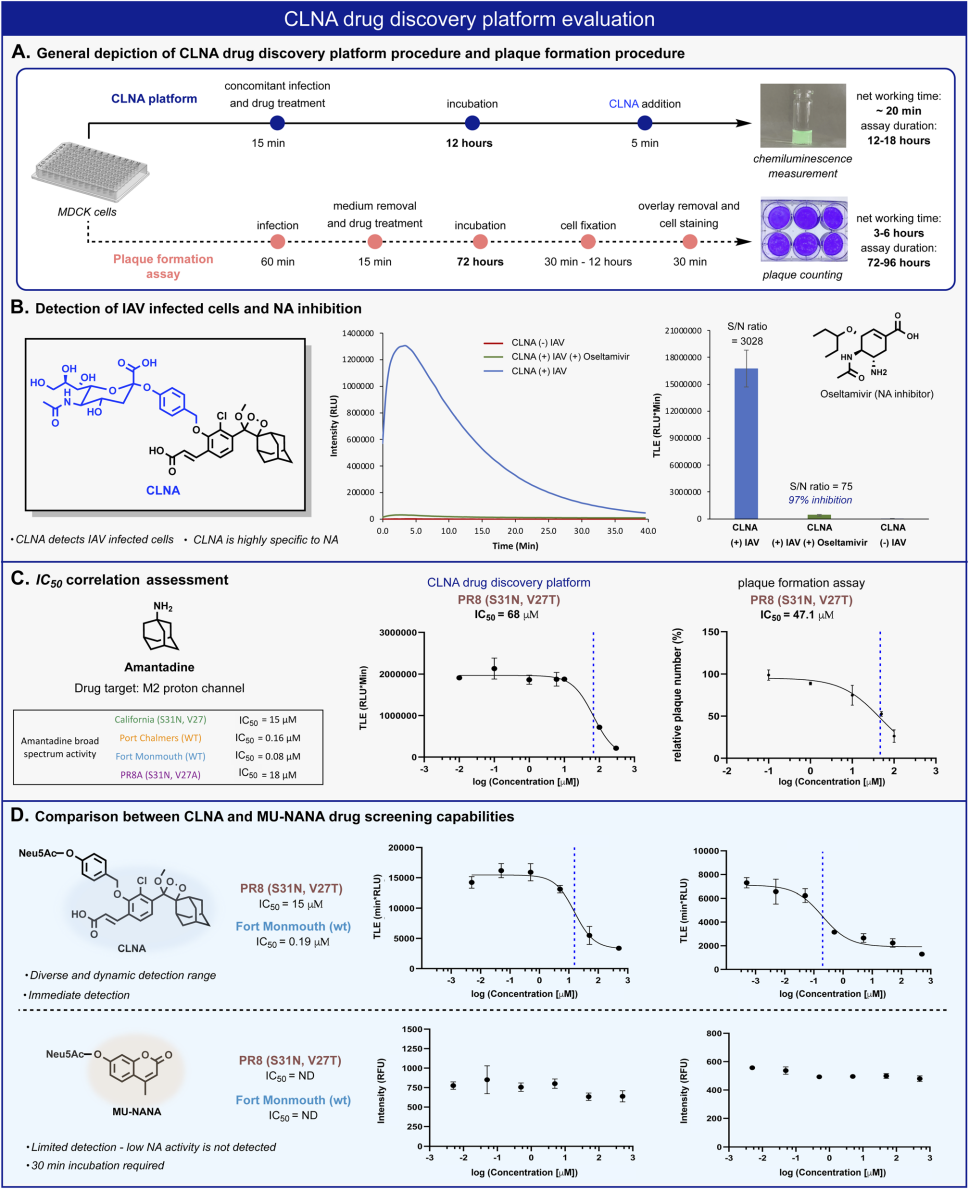
(A) Comparison between CLNA and plaque-formation assay workflow procedures. (B) Chemiluminescent kinetic profile (left) and total light emitted (right) from CLNA [10 μM] during 20 min in the presence of IAV-infected MDCK cells in 0.1% DMSO in PBS, pH 7.4 at 37 °C with and without oseltamivir carboxylate [50 nM]. (C) CLNA drug screening platform and plaque formation assay calculated IC_50_ values and sigmoidal fits for amantadine. The sigmoidal fits of the broad-spectrum evaluation are presented in the ESI.† (D) Relative intensity signal *vs.* log amantadine concentration of CLNA [10 μM] and MU-NANA [10 μM] (see ESI for detailed procedure†).

*In vitro* antiviral assays in cell-based systems are commonly performed by analysis of virus-mediated plaque formation. For viruses that form plaques in a cell monolayer, reduction in plaque formation is regarded as the ‘gold standard’ assay.^5^ We thus compared our CLNA-based chemiluminescence assay with a plaque formation assay (Fig. 4A). The neuraminidase activity was readily detected in IAV-infected Madin–Darby Canine Kidney (MDCK) cells using probe CLNA, with S/N of more than 3000-fold (Fig. 4B). To confirm that no viral or cellular activities other than neuraminidase contribute to the signal observed, CLNA was incubated with the NA inhibitor oseltamivir (a known FDA-approved drug for IAV). Oseltamivir inhibited 97% of the chemiluminescence signal produced by CLNA (Fig. S11†).

The chemiluminescence assay with CLNA should be able to detect the antiviral activity of small molecules that act through different mechanistic pathways. To confirm this, we performed the chemiluminescence assay in the presence of the antiviral drugs amantadine and arbidol, which inhibit viral replication by two different mechanisms.^45,50^ Amantadine targets the M2 proton channel, whereas arbidol targets envelope glycoprotein HA. IAV-infected MDCK cells were treated with various concentrations of each drug, followed by the addition of probe CLNA. The IC_50_ values obtained within 15 minutes post probe addition matched previously reported values for the tested IAV strains (Fig. 4C and S10†).^51^

In our hands, the IC_50_ value for amantadine as calibrant, obtained using a plaque formation assay was similar to that obtained using the CLNA-based assay. However, the plaque-based assay was much more labor intensive and time-consuming than the chemiluminescence assay: 72–96 hours for the plaque assay *vs.* 12–18 hours for the CLNA-based assay. Next, we demonstrated the advantage of the neuraminidase chemiluminescence assay in testing the activity of drug-like molecules for a broad-spectrum panel of IAVs. As a representative example, amantadine activity was evaluated against four additional strains (Fig. 4C, table) Similarly, measurements and analysis of amantadine IC_50_ values were rapidly obtained and shown to match literature values.^51^

To demonstrate the utility of the chemiluminescent CLNA assay as a drug screening platform, amantadine activity was evaluated in IAV-infected cells using a fluorescent MU-NANA-based assay and the CLNA-based assay. The cells were infected with multiplicity of infection (MOI) of 1.75 × 10^−4^ (PFU per cell), amantadine was added, and, after incubation, samples were tested using both assays. The IC_50_ value in the CLNA-based assay was expected based on the literature, whereas no inhibitory activity was detected using the MU-NANA-based assay (Fig. 4D). Considering the disadvantages of a MU-NANA-based assay mentioned above (low sensitivity and prolonged measurement duration), a reliable, high-throughput, NA-based assay is yet to be reported.

**Fig. 5:**
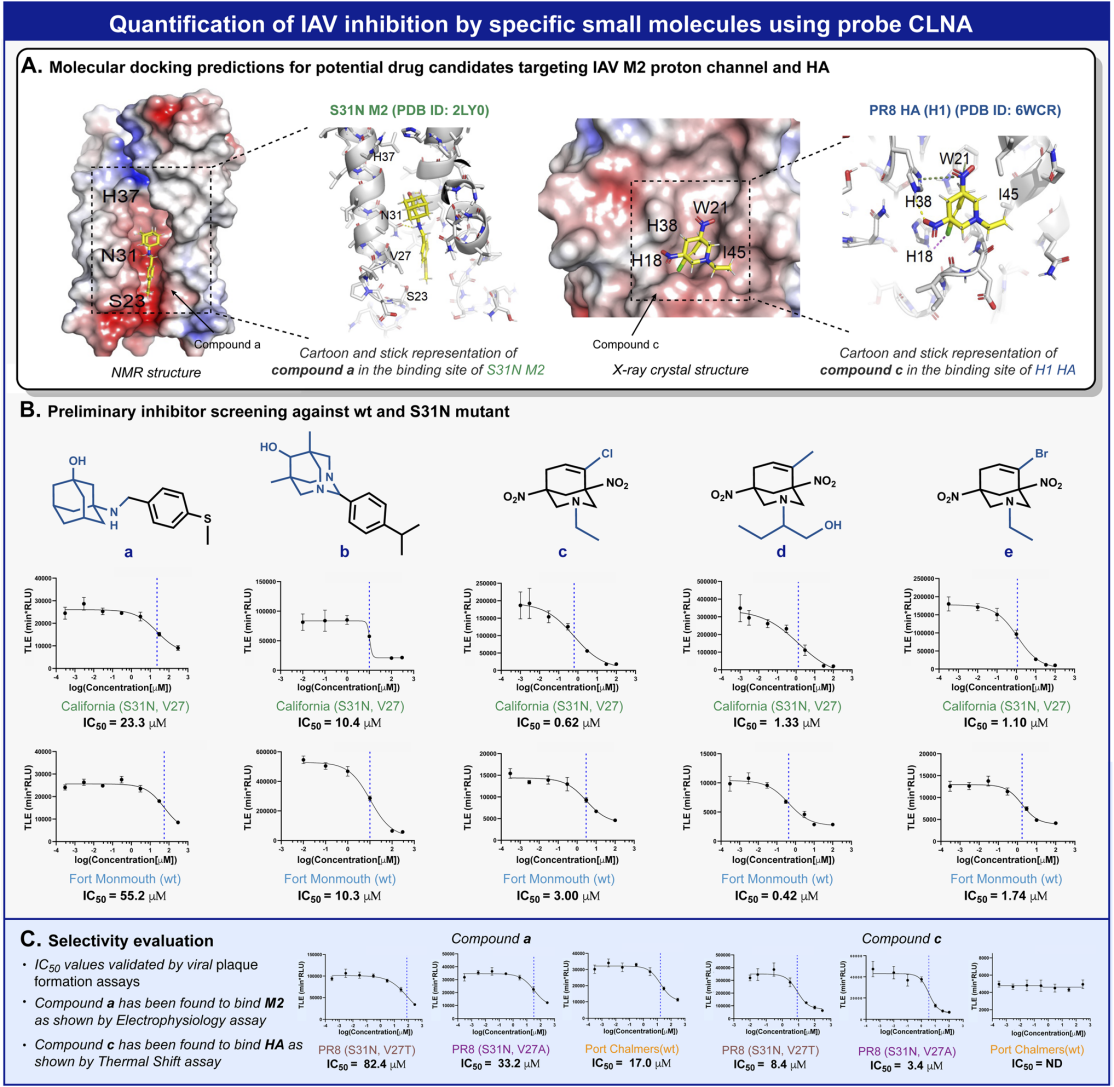
(A) Docking predictions of compound a to the NMR structure of mutant M2 from the California strain (left) and compound c to the crystal structure of PR8 H1 HA protein (right). (B) MDCK cell-based screening of five new drug candidates against FM and California IAV strains. (C) Broad spectrum activity evaluation for compound a and compound c.

Amantadine blocks the M2 proton channel of susceptible influenza viruses, thereby inhibiting viral replication. However, the emergence and spread of escape mutants that harbor mutations in the M2 viral channel have rendered this drug obsolete. In a search for new small drug-like molecules, a structure-activity relationship) SAR (-driven approach based on previously reported M2 inhibitors and docking simulations were used for several compounds with adamantane and adamantane-like scaffolds (Fig. 5A).^52,53^ Using our neuraminidase chemiluminescence assay, nine small molecules (Fig. 5B, compounds a–e and Fig. S26, and S28,† compounds f–i) were evaluated for their inhibition potency of viral replication. The assay was performed with MDCK cells infected with Fort Monmouth (FM) or the California IAV strains. The former expresses an amantadine-sensitive M2 protein, whereas the M2 protein in the latter has a mutation (S31N) that makes it amantadine-resistant. The sigmoid curves and the IC_50_ values obtained are presented in Fig. 5B. Potential compounds active in the initial screen were further evaluated for their broad-spectrum activity against three additional IAV strains, the amantadine-resistant A/Puerto Rico/8/34 (H1N1) (PR8), its sub-variant – the PR8 Mount Sinai (containing mutations S31N and V27A in M2 and H1 hemagglutinin), and an IAV strain that possesses the same “WT” M2 channel as influenza virus A/Fort Monmouth/1/47 (H1N1) used in the initial screen, but with a different HA subtype (H3) *i.e.*, A/Port Chalmers/1/73 (H3N2). In addition, electrophysiology and differential scanning fluorimetry (DSF) assays were performed to probe the targeted protein by the hit compounds and to provide a viable hypothesis for the engagement of these molecules with the respective protein.

To validate the molecular mechanism underlying viral replication inhibition of the amantadine-resistant strain by the drug-like molecules, we tested the ability of compound a to block the current produced by the A/California/07/2009 (H1N1) strain M2 viral channel. Following expression in *Xenopus* oocysts, proton currents were elicited by incubating the cells in an acidic environment (pH 5.5), containing 100 μM of inhibitors (Fig. S29†). In agreement with the CLNA-based amantadine inhibition results (Fig. 4C), incubating the cells with amantadine or compound a resulted in significant current inhibition (14.99 ± 1.53% and 28.71 ± 1.18%, respectively). Moreover, as deemed by both the CLNA assay and electrophysiological assay, compound a is not advantageous in blocking the M2 viral channel and in its potency in IVA-infected cells. Unexpectedly, compound c, showing the highest potency in the CLNA assay (Fig. 5B), did not produce M2 current inhibition (Fig. S30†). However, many adamantane-like compounds inhibit IAVs by interfering with HA function.^46,47^ Therefore, we proceeded with evaluating the ability of compound c to interact with HA, an envelope protein of IAV and a well-known drug target, using DSF.^54^ DSF was previously used to demonstrate the stabilization of HA's trimeric organization by the small-molecule antiviral *tert*-butyl hydroquinone (TBHQ), a known HA inhibitor that increases the energy barrier for the conformational change required for HA-mediated fusion.^55^ Accordingly, we hypothesized that significant differences in the denaturation profile of HA might occur upon drug binding. Compared with a DMSO control, incubation with 100 μM compounds c altered the denaturation profile of HA (Fig. S31†), significantly increasing the Sypro orange fluorescence ratio (*F*_41°C_/*F*_30°C_ = 0.29 ± 0.02 *vs.* 0.43 ± 0.02, for DMSO and compound c, respectively). Notably, compound i, a close derivative of compound c, exhibited a similar Sypro orange fluorescence ratio increase (*F*_41°C_/*F*_30°C_ = 0.38 ± 0.01), indicating the common ability of this scaffold to interact with HA (Fig. S31†). In contrast, compound a, which does not share this scaffold and includes an adamantane moiety, did not affect the thermal stability of HA (Fig. S32†). Together, these data confirm the use of the CLNA assay as a viable and sensitive choice for anti-IAV drug screening campaigns targeting the three major envelope proteins of IAVs.

Since our discovery that incorporating an acrylate substituent on a phenoxy-dioxetane luminophore dramatically improves the light-emission intensity in water, numerous similar chemiluminescence probes were evaluated for detection of various enzymes and bioanalytes. Although most of these probes showed high detection sensitivity with large S/N, probe CLNA is the most effective and sensitive chemiluminescence probe ever prepared, as a result of its high hydrolytic stability and excellent substrate's suitability towards its designated enzyme – the viral neuraminidase. The high hydrolytic stability resulted in almost no background signal and an S/N of more than 27 000-fold. Remarkably, probe CLNA also showed clear detection superiority compared to an analogous fluorescence probe (1000-fold higher LOD value) and an analogous chemiluminescence dioxetane probe (3000-fold higher LOD value), towards detection of IAV particles. The excellent sensitivity of CLNA enabled its use in a cell-based screen for inhibitors for viral replication regardless of drug mechanism of action. Since sialidases are involved in the pathogenesis of numerous infectious agents such as bacteria (*Vibrio cholerae*, *Streptococcus pneumonia*, *Pseudomonas aeruginosa*), viruses (Parainfluenza viruses, Newcastle disease virus, mumps virus), and various species of trypanosoma parasites, we envision that CLNA could be used in highly sensitive cell-based screening for drug candidates for treatment of these pathogens.^56^ It should be noted that although, there are two known chemiluminescence neuraminidase probes (NA-Star and ZstatFlu), CLNA is the only available probe that can be used under physiological conditions as a sole component for monitoring viral replication in mammalian cells.

## Conclusions

In summary, we have developed a new chemiluminescence probe for direct detection of neuraminidase activity. The probe activation mechanism is based on catalytic cleavage of a sialic acid substrate, followed by the release of phenoxy-dioxetane luminophore that undergoes an efficient chemiexcitation process to emit a green photon. The probe exhibits an effective turn-on response upon reaction with neuraminidase and produces an intense light emission signal with an extremely high signal-to-noise ratio. Comparing the new dioxetane probe with analogous fluorescence and chemiluminescence probes showed superior detection capability regarding response time and sensitivity, making it the most sensitive neuraminidase probe known to date. The chemiluminescence turn-on response produced by the neuraminidase probe enabled rapid screening for small molecules that inhibit viral replication through different mechanism. The screening assay is directly performed in influenza A-infected mammalian cells. We expect that our new chemiluminescence neuraminidase probe CLNA will be useful for various applications requiring neuraminidase detection, including drug discovery assays in mammalian cells against various influenza virus strains and other pathogens.

## Author contributions

O. S. and S. G. performed the synthesis, analyzed CLNA assay data under the supervision of D. S., and contributed equally, D. F. performed the computational simulation under the supervision of N. B. -T., D. F. conducted the viral plaque formation assays, and prepared viral related experiments under the supervision of E. B., A. B. -B. performed electrophysiology and DSF assays under the supervision of Y. H., all authors contributed to the discussion and revision of the manuscript.

## Conflicts of interest

There are no conflicts to declare.

## Supplementary Material

SC-013-D2SC03460C-s001
