# Supplementary material for: Ultrasensitive chemiluminescent neuraminidase probe for rapid screening and identification of small-molecules with antiviral activity against influenza A virus in mammalian cells
Source: Chem Sci. 2022 Sep 26;13(42):12348–57. doi: 10.1039/d2sc03460c (PMC9629042; doi:10.1039/d2sc03460c)
Supplement: SC-013-D2SC03460C-s001 [file SC-013-D2SC03460C-s001.pdf]

Electronic Supplementary Material (ESI) for Chemical Science.
This journal is © The Royal Society of Chemistry 2022

Ultrasensitive Chemiluminescent Neuraminidase Probe for Rapid Screening and Identification of Small-molecules with Antiviral Activity Against Influenza A Virus in Mammalian Cells

Omri Shelef[a,#], Sara Gutkin[a,#], Daniel Feder[b,c,#], Ariel Ben-Bassat[d], Michal Mandelboim[e,f], Yoni Haitin[d], Nir Ben-Tal[b], Eran Bacharach[c], and Doron Shabat[a]*

[a]School of Chemistry, Raymond and Beverly Sackler Faculty of Exact Sciences, Tel-Aviv University, Tel Aviv 69978 Israel.

[b]School of Neurobiology, Biochemistry and Biophysics, George S. Wise Faculty of Life Sciences, Tel Aviv University, Tel Aviv 69978 Israel.

[c]The Shmunis School of Biomedicine and Cancer Research, George S. Wise Faculty of Life Sciences, Tel Aviv University, Tel Aviv 69978 Israel.

[d]Department of Physiology and Pharmacology, Sackler Faculty of Medicine, Tel Aviv University, Tel Aviv, Israel

[e]Central Virology Laboratory, Sheba Medical Center, Tel Hashomer, Ramat-Gan 52620 Israel.

[f]School of Public Health, Sackler Faculty of Medicine, Tel Aviv University, Tel Aviv 69978 Israel

[#]These authors contributed equally

Supporting Information

Table of Contents

General methods S3

Synthesis and characterization of **CLNA** S4

Viral Experiments S8

Supplementary Figures S11

NMR spectra S32

MS and/or HPLC Spectra of Key Compounds S40

References S40

General methods

All reactions requiring anhydrous conditions were performed under an Argon atmosphere. All reactions were carried out at room temperature unless stated otherwise. Chemicals and solvents were either A.R. grade or purified by standard techniques. Thin-layer chromatography (TLC): silica gel plates Merck 60 F254: compounds were visualized by irradiation with UV light. Column chromatography (FC): silica gel Merck 60 (particle size 0.040-0.063 mm), eluent given in parentheses. Reverse-phase high-pressure liquid chromatography (RP-HPLC): C18 5u, 250x4.6mm, eluent given in parentheses. Preparative RP-HPLC: C18 5u, 250x21mm, eluent given in parentheses. ^{1}H-NMR spectra were measured using Bruker Avance operated at 400MHz. ^{13}C-NMR spectra were measured using Bruker Avance operated at 100 MHz. Chemical shifts were reported in ppm on the δ scale relative to a residual solvent ($CDCl_3$: δ = 7.26 for ^{1}H-NMR and 77.16 for ^{13}C-NMR, DMSO-d_6: δ = 2.50 for ^{1}H-NMR and 39.52 for ^{13}C-NMR). Mass spectra were measured on Waters Xevo TQD. Chemiluminescence was recorded on Molecular Devices Spectramax i3x. Fluorescence was recorded on Tecan infinite 200 Pro. All general reagents, including salts and solvents, were purchased from Sigma-Aldrich. Light irradiation for photochemical reactions: LED PAR38 lamp (19W, 3000K).

Abbreviations

ACN- Acetonitrile, **$CHCl_3$**- Chloroform, **DCM**- dichlorometane, **DIPEA**- N,N-Diisopropylethylamine, **DMF**- N,N' –Dimethylformamide, **EtOAc**- Ethylacetate, **Hex**-Hexanes, **iPrOH**- Isopropyl alcohol, **K_2CO_3**$^-$ Potassium carbonate, **LiOH**- Lithium hydroxide **MB**- Methylene blue **MeOH**- Methanol, **NH_4Cl**- ammonium chloride, **$NaHCO_3$**$^-$ Sodium bicarbonate, **$Na_2S_2O_3$**$^-$ Sodium Thiosulfate, **Na_2SO_4**$^-$ Sodium Sulfate, **$NaBH_4$**- Sodium borohydride, **NaI**- Sodium Iodide, **THF**- Tetrahydrofuran, **TMS-Cl** -Trimethylsilyl chloride, **DIPEA**- Diisopropylethyl amine.

Synthesis and characterization of CLNA

Procedures

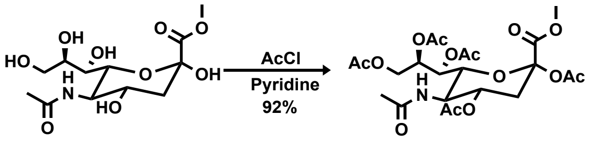

1

Compound 1

N-acetylneuraminic acid methyl ester (1 gr, 3.1 mmol, 1 eq) was dissolved in 5 ml of pyridine and cooled to 0°C. Acetyl chloride (1.76 ml, 24 mmol, 8 eq) was added dropwise, and upon completion the reaction mixture was stirred over night at room temperature. Afterwards reaction mixture was diluted with EtOAc and washed with HCl 1M. The organic layer was separated, dried over Na_2SO_4, filtered and the solvent was evaporated under reduced pressure, to afford compound 1 (1.5 gr, 92% yield) as an off-white solid. The compound was reacted without further purification. MS (ES+): m/z calc. for $C_{22}H_{31}NO_{14}$: 533.17; found: 556.4 $[M+Na]^+$. ^{1}H NMR (400 MHz, DMSO) δ 5.62-5.64 (m, 1H), 5.35-5.37 (m, 1H), 5.22 (m, 1H), 5.04 (m, 1H), 4.48 (dd, *J* = 2.5, 12.5 Hz, 1H), 4.09-4.13 (m, 3H), 3.77 (s, 3H), 2.52 (dd, *J* = 4.9, 13.5 Hz, 1H), 2.20 (s, 3H), 2.13 (s, 6H), 2.01 (s, 6H), 1.87 (s, 3H), 1.23 (t, *J* = 7.1 Hz, 1H). ^{13}C NMR (100 MHz, DMSO) δ 171.14, 170.75, 170.59, 170.52, 170.43, 168.39, 166.49, 67.61, 72.94, 71.66, 68.48, 68.02, 62.28, 53.30, 49.38, 36.05, 23.20, 21.00, 20.94, 20.86

Compound 2

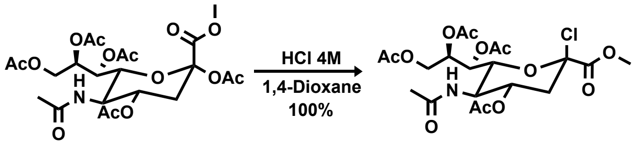

1 2

Hydrochloric acid 4M in dioxane (2 ml) was add to compound 1 (210mg, 0.39 mmol) under argon atmosphere. Reaction was monitored by LC-MS and Upon completion was concentrated by evaporation under reduced pressure to afford compound 2 (200 mg, 100% yield) as a colorless oil. The compound was reacted without further purification. MS (ES+): m/z calc. for $C_{20}H_{28}NO_{12}$: 509.13; found: 510.4 $[M+H]^+$. ^{1}H NMR (400 MHz, $CDCl_3$) δ 5.38 – 5.35 (m, 1H), 5.34 – 5.21 (m, 1H), 5.07 (ddd, *J* = 6.8, 5.2, 2.6 Hz, 1H), 4.49 (dd, *J* = 12.4, 2.6 Hz, 1H), 4.12 (M, *J* = 9.3, 6.0, 2.4 Hz, 4H), 3.79 (s, 3H), 2.55 (dd, *J* = 13.5, 5.0 Hz, 2H), 2.14 (s, 3H), 2.14 (s, 3H), 2.09 (s, 3H), 2.06 (s, 3H), 1.89 (s, 3H). ^{13}C NMR (101 MHz, $CDCl_3$) δ 171.35, 171.13, 171.04, 170.33, 170.31, 165.85, 73.80, 70.41, 69.04, 67.18, 67.13, 62.26, 53.83, 48.36, 40.65, 22.72, 20.92, 20.82, 20.75, 20.68.

Compound 3

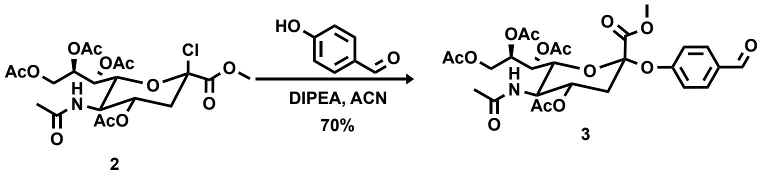

DIPEA (327μL, 1.88 mmol, 3 eq) was added to Compound 2 (320mg, 0.62mmol, 1 eq) and 4-Hydroxybenzaldehyde (153 mg, 1.25 mmol, 2 eq) dissolved in ACN. The reaction mixture was stirred at room temperature and monitored by TLC (EtOAc). Upon completion, the reaction mixture was diluted with EtOAc, and washed with brine. The organic layer was separated, dried over Na_2SO_4, filtered and the solvent was evaporated under reduced pressure. The crude product was purified by column chromatography on silica gel (EtOAc) to afford compound 3c (234 mg, 58% yield) as a white solid. MS (ES+): m/z calc. for $C_{27}H_{33}NO_{14}$: 595.19; found: $[M+Na]^+$ ^{1}H NMR (400 MHz, $CDCl_3$) δ 9.91 (s, 1H), 7.82 (d, *J* = 8.8 Hz, 2H), 7.16 (d, *J* = 8.7 Hz, 2H), 5.41 (d, *J* = 10.3 Hz, 1H), 5.39 – 5.26 (m, 2H), 4.97 (ddd, *J* = 12.1, 10.4, 4.6 Hz, 1H), 4.65 – 4.53 (m, 1H), 4.42 – 4.33 (m, *J* = 1.8 Hz, 1H), 4.15 – 4.06 (m, 2H), 3.64 (s, 3H), 2.72 (dd, *J* = 13.1, 4.7 Hz, 1H), 2.30 (dd, *J* = 25.8, 13.2 Hz, 1H), 2.17 (s, 3H), 2.10 (s, 6H), 2.08 – 2.00 (m, 3H), 1.91 (s, 3H). 13C NMR (101 MHz, CDCl3) δ 191.13, 171.05, 170.72, 170.48, 170.29, 170.16, 168.40, 159.12, 132.13, 131.80, 119.06, 99.67, 73.78, 70.82, 68.92, 68.52, 67.98, 67.32, 62.18, 53.31, 49.50, 38.75, 23.31, 21.12, 20.95, 20.84, 20.84.

Compound 4

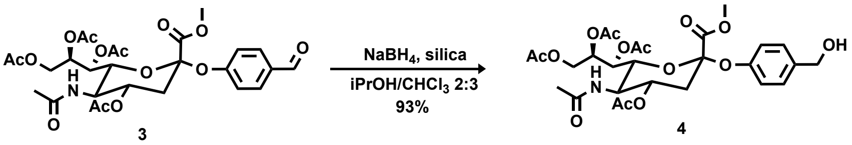

Compound 3 (140 mg, 0.23 mmol, 1 eq) and silica gel (43 mg, 0.7 mmol, 3 eq) were dissolved in 2 ml in a mixture of iPrOH and $CHCl_3$ (2:3) and cooled to 0°C. $NaBH_4$ (9 mg, 0.23 mmol, 1 eq) was add to the reaction mixture and the reaction was allowed warm up to room temperature. The reaction mixture was stirred at room temperature and monitored by TLC (EtOAc). Upon completion, the reaction mixture was diluted with EtOAc (100 mL) and washed with 0.1M HCl (50 mL) and brine (50 mL). The organic layer was separated, dried over Na_2SO_4 and evaporated under reduced pressure to afford compound **4** (130 mg, 93% yield) as a off-white solid. Compound 4 was reacted without further purification. MS (ES+): m/z calc. for $C_{27}H_{35}NO_{14}$: 597.21; found:620.6 $[M+Na]^+$.^{1}H NMR (400 MHz, $CDCl_3$) δ 7.22 (d, *J* = 8.4 Hz, 2H), 6.99 (d, *J* = 8.4 Hz, 2H), 5.74 (d, *J* = 9.9 Hz, 1H), 5.46 (s, 1H), 4.90 (td, *J* = 11.8, 4.5 Hz, 1H), 4.58 (s, 3H), 4.34 (d, *J* = 10.9 Hz, 2H), 4.25 (dd, *J* = 12.4, 2.0 Hz, 1H), 4.19 – 4.12 (m, 1H), 3.63 (s, 3H), 2.67 (dd, *J* = 12.9, 4.5 Hz, 1H), 2.17 (d, *J* = 12.5 Hz, 1H), 2.11 – 2.04 (m, *J* = 6.4, 3.5 Hz, 9H), 1.85 (t, *J* = 7.1 Hz, 3H), 1.37 (s, 1H), 1.25 – 1.16 (m, 3H). ^{13}C NMR (101 MHz, $CDCl_3$) δ 171.07, 170.88, 170.73, 170.41, 170.20, 168.27, 161.76, 153.09, 145.21, 136.91, 128.31, 120.37, 108.19, 100.04, 73.46, 69.71, 68.99, 62.24, 60.54, 52.65, 49.37, 46.56, 38.17, 23.18, 20.94, 20.83, 20.82.

Compound 5

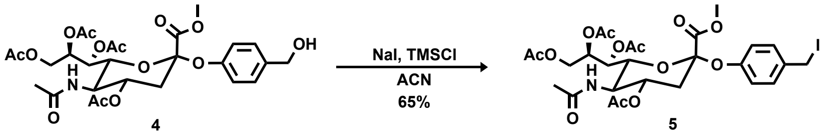

Compound 4 (86 mg, 0.14 mmol, 1 eq) was dissolved in 1 ml of ACN and cooled to 0 °C. Sodium Iodide (64 mg, 0.43 mmol, 3 eq) was added followed by the rapid addition of TMS-Cl (54 µl, 0.43 mmol, 3 eq). The reaction was allowed to warm up to room temperature and monitored by TLC (EtOAc). Upon completion, the reaction mixture was diluted with EtOAc, and washed with saturated $Na_2S_2O_3$ followed by brine. The organic layer was separated, dried over Na_2SO_4, filtered and the solvent was evaporated under reduced pressure, the crude product was purified by column chromatography on silica gel (EtOAc) to afford the compound 5 (70 mg, 65% yield) an off-white solid. MS (ES+): m/z calc. for $C_{27}H_{34}INO_{13}$: 707.11; found:708.5 $[M+H]^+$. ^{1}H NMR (400 MHz, $CDCl_3$) δ 7.26 (d, *J* = 6.5 Hz, 1H), 6.95 (d, *J* = 8.1 Hz, 2H), 5.52 – 5.42 (m, *J* = 11.8 Hz, 2H), 5.34 (s, 3H), 4.97 – 4.86 (m, 1H), 4.61 (d, *J* = 12.2 Hz, 1H), 4.41 (s, 3H), 4.29 (d, *J* = 12.1 Hz, 1H), 3.63 (s, 3H), 2.67 (dd, *J* = 12.9, 4.1 Hz, 1H), 2.19 (t, *J* = 12.7 Hz, 1H), 2.13 (s, 3H), 2.10 (s, 6H), 1.90 (s, 3H), 1.89 (s, 3H). ^{13}C NMR (101 MHz, $CDCl_3$) δ 171.06, 170.75, 170.48, 170.30, 170.16, 168.27, 153.45, 145.22, 134.78, 130.00, 120.02, 108.08, 99.99, 73.50, 70.90, 69.32, 68.85, 67.79, 62.14, 53.12, 49.48, 46.69, 38.27, 23.29, 23.23, 20.96, 20.86.

Compound 6

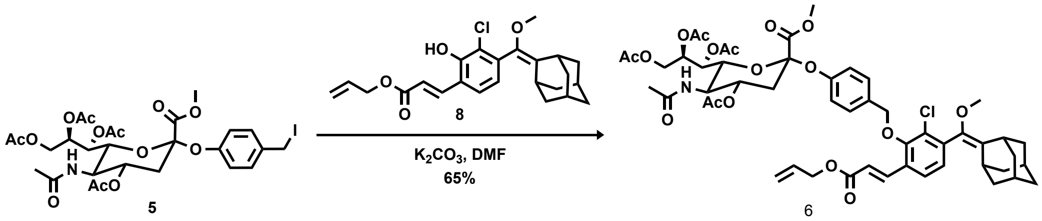

Phenol enol ether 8[2] (50 mg, 0.12 mmol, 1.2 eq) and K2CO3 (26 mg, 0.18 mmol, 2 eq) were dissolved in DMF (1 mL). The solution was stirred for 5 minutes before compound 5 (80 mg, 0.11 mmol, 1 eq) was added. The reaction mixture was stirred at room temperature and monitored by TLC (EtOAc). Upon completion, the reaction mixture was diluted with EtOAc (100 mL) and washed with 0.1M HCl (50 mL) and brine (50 mL). The organic layer was separated, dried over Na2SO4 and evaporated under reduced pressure. The crude product was purified by column chromatography on silica gel (EtOAc). Compound 6 was obtained as a white solid (72 mg, 65% yield). MS (ES-): m/z calc. for C51H60ClNO17: 993.35; found: 992.8 $[M+H]^-$. 1H NMR (400 MHz, $CDCl_3$) δ 8.01 (d, *J* = 16.2 Hz, 1H), 7.46 (dd, *J* = 13.1, 8.3 Hz, 3H), 7.12 – 7.03 (m, 3H), 6.51 (d, *J* = 16.2 Hz, 1H), 6.08 – 5.96 (m, 2H), 5.43 – 5.36 (m, 2H), 5.34 – 5.26 (m, 2H), 5.01 – 4.94 (m, 3H), 4.74 (d, *J* = 5.6 Hz, 1H), 4.46 (d, *J* = 10.8 Hz, 1H), 4.36 – 4.29 (m, 1H), 4.23 – 4.16 (m, 1H), 3.69 (s, 3H), 3.35 (s, 3H), 3.30 (s, 1H), 2.72 (dd, *J* = 13.0, 4.6 Hz, 1H), 2.25 (t, *J* = 12.6 Hz, 1H), 2.17 (s, 3H), 2.15 (s, 3H), 2.11 – 2.08 (m, 2H), 2.06 (s, 6H), 1.93 (s, 3H), 1.89 – 1.74 (m, 6H), 1.69 – 1.66 (m, 4H). ^{13}C NMR (101 MHz, DMSO) δ 170.58, 170.25, 170.03, 169.77, 168.38, 166.12, 154.20, 153.56, 145.32, 139.94, 138.50, 138.08, 133.24, 131.66, 130.91, 130.55, 129.89, 129.22, 128.33, 126.42, 120.75, 119.80, 118.49, 109.13, 100.22, 76.62, 75.70, 73.34, 69.48, 69.16, 68.71, 67.53, 65.24, 62.27, 60.33, 57.14, 53.63, 52.94, 48.36, 38.97, 38.71, 38.35, 37.05, 33.02, 29.64, 28.17, 23.17, 21.30, 21.15, 21.10, 14.67.

Compound 7

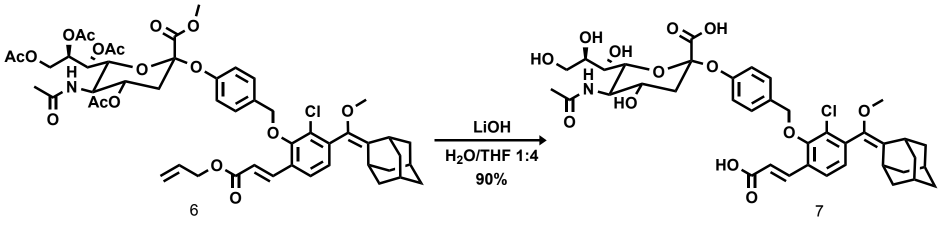

Compound 6 (72 mg, 0.07 mmol, 1 eq) and LiOH (17 mg, 0.7 mmol, 10 eq) were dissolved in 3 mL solution of 4:1 THF:H2O. Reaction mixture was stirred at 60 °C and monitored by RP-HPLC. Upon completion, the solvent was concentrated under reduced pressure and the product was purified by preparative RP-HPLC (gradient of ACN in water). Compound **7** was obtained as a white solid (50 mg, 90% yield). MS (ES+): m/z calc. for $C_{51}H_{60}ClNO_{17}$: 771.27; found:794.6 $[M+Na]^+$. ^1H NMR (400 MHz, DMSO) δ 9.05 (s, 1H), 8.10 – 8.03 (m, 2H), 7.77 (dd, *J* = 12.1, 4.0 Hz, 2H), 7.37 (d, *J* = 8.4 Hz, 2H), 7.18 – 7.08 (m, *J* = 18.1, 8.1 Hz, 2H), 6.58 (d, *J* = 16.2 Hz, 1H), 5.16 – 5.05 (m, 1H), 4.92 – 4.82 (m, 2H), 4.67 – 4.59 (m, 1H), 4.36 – 4.21 (m, *J* = 25.1, 12.4 Hz, 1H), 3.74 (d, *J* = 10.0 Hz, 1H), 3.65 – 3.51 (m, 2H), 3.21 (s, 3H), 3.17 (s, 1H), 2.58 (dd, *J* = 12.3, 3.8 Hz, 1H), 2.16 (t, *J* = 7.3 Hz, 1H), 2.00 – 1.94 (m, 2H), 1.92 – 1.85 (m, *J* = 6.5 Hz, 3H), 1.80 – 1.68 (m, 2H), 1.35 – 1.15 (m, 14H), 0.86 – 0.79 (m, 1H). ^{13}C NMR (101 MHz, DMSO) δ 172.59, 172.34, 170.03, 167.90, 154.71, 153.51, 139.98, 137.74, 137.63, 131.68, 130.90, 130.09, 130.09, 129.24, 128.37, 126.41, 122.39, 121.20, 100.97, 74.99, 71.76, 69.24, 66.73, 63.99, 57.15, 52.75, 38.69, 37.05, 33.02, 32.13, 30.02, 29.58, 29.27, 28.29, 28.15, 23.26, 22.67, 21.64, 14.53.

CLNA

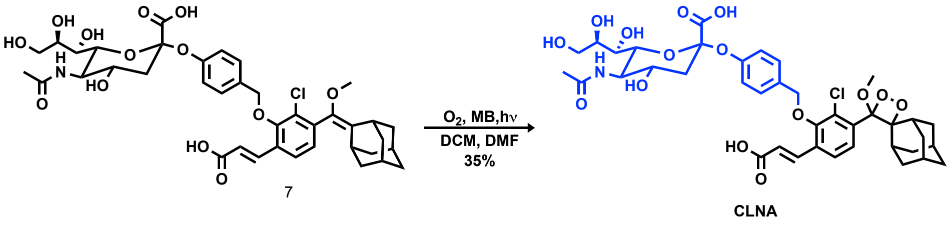

Compound 7 (30 mg, 0.04 mmol) and a catalytic amount of methylene blue (~1 mg) were dissolved in 10 mL of DCM. Oxygen was bubbled through the solution while irradiating with yellow light. The reaction was monitored by RP-HPLC. Upon completion, the solvent was concentrated under reduced pressure and the product was purified by preparative RP-HPLC (gradient of ACN in water). **CLNA** was obtained as a white solid (8 mg, 26% yield). MS (ES-): m/z calc. for $C_{36}H_{46}ClNO_{15}$: 803.26; found:802.6 $[M-H]^-$ ^1H NMR (400 MHz, DMSO) δ 8.03 – 7.92 (m, 2H), 7.79 – 7.70 (m, 1H), 7.39 (d, *J* = 8.5 Hz, 2H), 7.16 (d, *J* = 8.6 Hz, 2H), 6.64 (d, *J* = 16.1 Hz, 1H), 4.83 (dd, *J* = 17.5, 10.6 Hz, 2H), 3.87 (s, 1H), 3.75 (d, *J* = 9.1 Hz, 2H), 3.67 – 3.55 (m, 4H), 3.11 (s, 3H), 2.88 (s, 1H), 2.58 (dd, *J* = 12.5, 3.7 Hz, 1H), 2.23 (d, *J* = 10.8 Hz, 1H), 2.02 (d, *J* = 10.8 Hz, 1H), 1.92 – 1.83 (m, 4H), 1.81 – 1.43 (m, 12H), 1.37 – 1.28 (m, 1H), 1.20 (d, *J* = 10.7 Hz, 1H). ^{13}C NMR (101 MHz, DMSO) δ 172.26, 170.06, 167.70, 154.73, 154.19, 137.14, 134.72, 131.93, 131.52, 130.19, 129.06, 127.40, 126.65, 123.67, 121.21, 111.79, 100.88, 96.04, 75.85, 75.03, 71.73, 69.24, 66.72, 64.01, 52.67, 50.02, 41.31, 39.06, 36.47, 33.89, 33.68, 32.44, 32.25, 31.70, 31.48, 26.10, 25.76, 23.27.

Viral Experiments

Virus preparation and titration

Virus stocks were prepared in MDCK cell cultures. The viruses used were A/PR/08/34 (H1N1), A/Fort Monmouth/1/1947 (H1N1), A/Port Chalmers/1/73 (H3N2), A/California/07/2009 (H1N1) (all purchased from ATCC™), and A/PR/8/34 Mount Sinai (H1N1) subvariant, provided by Dr. Michal Mandelboim lab. A confluent monolayer of MDCK cells was incubated with DMEM (Gibco™) containing 2% fetal bovine serum (FBS), 0.6 µg/ml trypsin from porcine pancreas (T0303; Sigma Aldrich™), Pen-Strep-Neomycin solution (PSN) and virus at 0.01 MOI at 37° C 5% CO_2. After 72 hours, the supernatant was aliquoted and stored at -80°C. The titer of the virus was determined by plaque assay. First, by incubation of a confluent layer of MDCK cells in 6-well plates with 750 µl DMEM containing 10-fold dilutions of virus stocks, supplemented with 0.6 µg/ml T0303, which were incubated at 37° C 5% CO_2 for 1 hour, with rocking every 15 minutes, followed by placement of an agarose overlay of DMEM (2 % FBS, PSN, 0.45% Seakem agarose) and incubation at 37° C 5% CO_2 for 72 hours. Plaque formation indicated the presence of a single plaque-forming unit, and the amount of plaque-forming units per milliliter (PFU/ml) was subsequently calculated.

Plaque assay with antivirals

Confluent monolayers of MDCK cells in 6-well plates (in triplicates) were incubated with 750 µl DMEM (Gibco™) containing 0.6 µg/ml trypsin T0303 and ~100 PFU of virus for 1 hour at 37° C, 5% CO_2, rocking the plate every 15 mins, followed by placement of an agarose overlay of DMEM (2 % FBS; 0.6 µg/ml T0303; 0.45% Seakem™ agarose) containing 10-fold dilutions of antiviral compound/s. After 72 hours, 1 ml of PFA was placed on each agarose overlay and allowed to fix the cells and plaques overnight at 4° C. para-Formaldehyde was then removed with a pipette, and the agarose plug was removed gently by vacuum. Fixed cells were washed with Dulbecco's™ phosphate-buffered saline (PBS) and a solution of 5% crystal violet containing 20% methanol was placed on top of the cells, followed by incubation at room temperature for ~30 mins. Stained cells were then washed with water and allowed to dry. Plaques were counted against a white backlight, and plaque reduction was calculated as % reduction in plaque number relative to the well that was untreated with the antiviral. Dose-response curves were plotted using GraphPad Prism™.

Antivirals

Amantadine (Sigma™ Chemicals, St Louis MO), Arbidol, and Oseltamivir (Sigma™ Chemicals) were dissolved in DMSO to make 100- and 200-mM stock solutions. All stock solutions were stored at -20° C. All novel inhibitors described here were purchased from ChemBridge (ChemBridge™, San Diego, CA). and, based on their solubility, were dissolved in DMSO to make 100- or 200-mM stock solutions.

CLNA Viral Inhibition Assay

Confluent monolayers of MDCK cells in 96-well plates (in tetraplicates) were incubated with 100 µl DMEM (Gibco™) containing 10 ng/ml trypsin T0303, 2 % FBS and ~7-70 PFU of virus (MOI = 1.75×10^{-4}) with 10-fold serial dilutions of antiviral compound/s ranging from 1 nM to 100 µM. After 12-18 hours, the media were aspirated and 50 µl phenol red free PBS, pH 7.4 (50 µl) (Sigma™), was added to each well, followed by the addition of another 50 µl PBS containing 10 µM CLNA (pre-warned to 37° C), 0.1% DMSO (final CLNA concentration was 5

µM in PBS, 0.1% DMSO). The plate was then placed in a Spectramax i3x spectrophotometer and subsequent measurement of chemiluminescent light emission intensity was immediately performed for a duration of 5-15 min at 37° C. Dose-response curves were plotted using GraphPad Prism™. Sigmoidal non-linear curves were used to calculate the IC_{50} of inhibitors.

MU-NANA Viral Inhibition Assay

Confluent monolayers of MDCK cells in 96-well plates (in tetraplicates) were incubated with 100 µl DMEM (Gibco™) containing 10 ng/ml trypsin T0303, 2 % FBS and ~7-70 PFU of virus (MOI = 1.75×10^{-4}) with 10-fold serial dilutions of antiviral compound/s ranging from 1 nM to 100 µM. After 12-18 hours, the media were aspirated and 50 µl phenol red free PBS, pH 7.4 (50 µl) (Sigma™), was added to each well, followed by the addition of another 50 µl PBS containing 10 µM MU-NANA (pre-warned to 37° C), 0.1% DMSO (final MU-NANA concentration was 5 µM in PBS, 0.1% DMSO). The plate was then placed in a Spectramax i3x spectrophotometer and subsequent measurement of fluorescent light emission intensity was immediately performed for a duration of 30-60 minutes at 37° C. Dose-response curves were plotted using GraphPad Prism™. Sigmoidal non-linear curves were used to calculate the IC_{50} of inhibitors.

Statistical analysis

Statistical analysis was performed using GraphPad Prism software, with sigmoidal non-linear curves used to calculate the IC50 of inhibitors.

Channel Expression into Xenopus Oocytes

Oocytes were obtained by surgical removal of ovary pieces from Xenopus laevis frogs (Xenopus 1) anesthetized with 0.15% tricaine (Sigma-Aldrich). The procedures for surgery and maintenance of frogs were approved by the animal research ethics committee of Tel Aviv University and in accordance with the Guide for the Care and Use of Laboratory Animals (National Academy of Sciences). Oocytes were de-folliculated using 1 mg/ml collagenase (type IA; Sigma-Aldrich) in Ca2+-free ND96 solution containing (in mM) 96 NaCl, 2 KCl, 1 MgCl2, and 5 HEPES, pH 7.5 (ND96), for ~1 h. Stage V and VI oocytes were selected for RNA injection and maintained at 18°C in ND96, supplemented with 1 mM pyruvate and 50 µg/ml gentamycin. Expression of Influenza A/California/07/2009 M2 proton channel was achieved by injecting 50 nl per oocyte of in vitro transcribed cRNA (New England Biolabs) (1.25 ng) using a Nanoject injector (Drummond).

Two-electrode voltage clamp

Standard two-electrode voltage-clamp measurements were performed at room temperature (22–24°C) 1 day after cRNA microinjections. Oocytes were placed into a 100 µl recording chamber and perfused with Barth's solution containing (in mM) 88 NaCl, 1 KCl, 2.4 NaHCO3, 0.3 NaNO3, 0.71 CaCl2, 0.82 MgSO4, 15 HEPES, pH 7.5, or 15 MES, pH 5.5. 100 µM of ligands were dissolved in pH 5.5 Barth's solution. Whole-cell currents were recorded using a GeneClamp 500 amplifier through the pCLAMP 9.2.1.9 software (Axon Instruments, Inc.). Glass microelectrodes (A-M Systems, Inc.) were filled with 3 M KCl and had tip resistances of 0.3–1 MΩ. Current signals were digitized at 250 Hz and low-pass filtered at 20 Hz. The holding potential was −20 mV.

Differential Scanning Fluorimetry

Differential Scanning Fluorimetry (DSF) was performed using a real-time polymerase chain reaction system (Thermo Fisher Scientific) with the fluorescent dye SYPRO Orange (Thermo Fisher Scientific) using the ROX filter set in clear 96-well plates[3]. The temperature was increased using a continuous ramp at a rate of 1 °C/min from 4 to 95°C. Assays were performed in a final volume of 25 µl, containing 1 µg influenza A H1N1 (A/California/07/2009) hemagglutinin (Sino Biological), 5X SYPRO Orange, dissolved in DSF buffer containing (in mM) 150 NaCl, 500 MES, pH 5.2, and 100 µM ligands (dissolved in DMSO).

Supplementary Figures

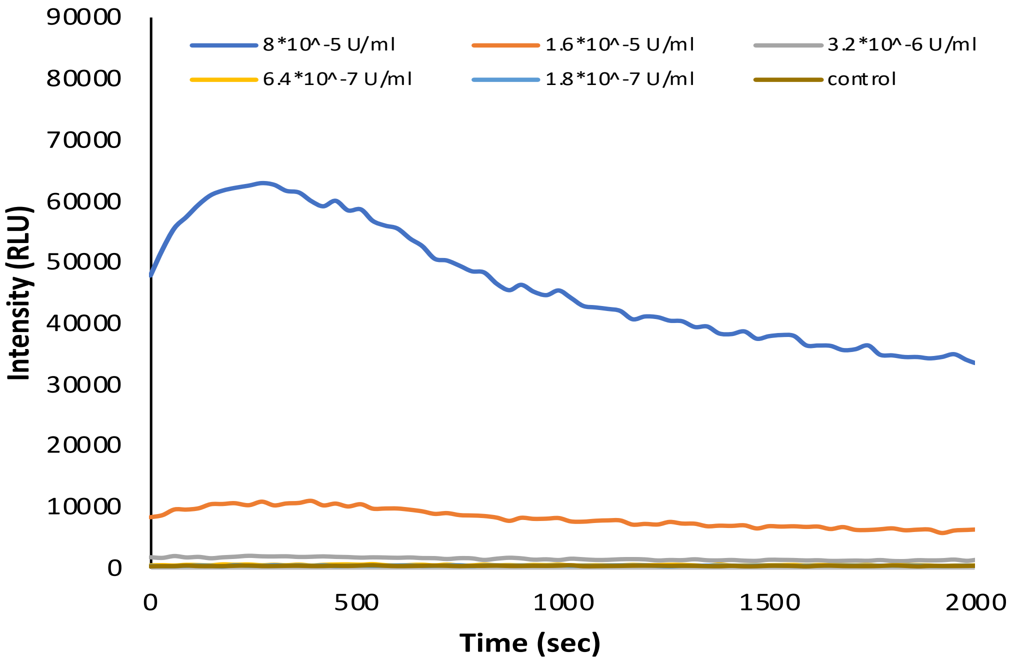

Figure S1. Chemiluminescence kinetic profile **CLNA** [10 µm], 1% DMSO in PBS 4mM CaCl2, in the presence and absence of different *Neuraminidase concentrations* (U/ml) (*from C. perfringens*), measured at 25° C.

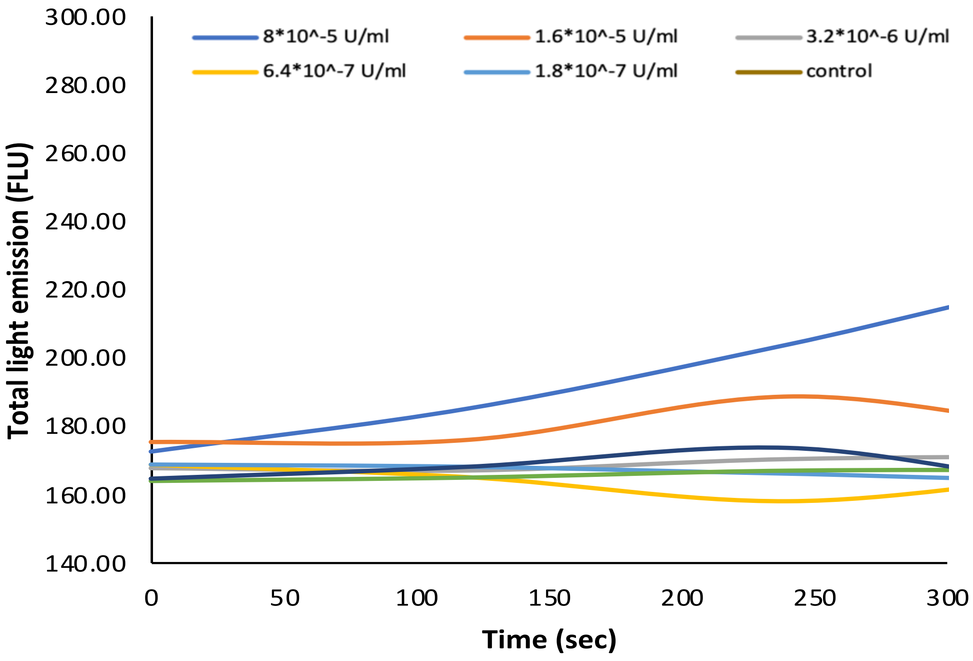

Figure S2. Total light emission plotted against time of MU-NANA [10 µm], 1% DMSO in PBS, CaCl2 [4mM] plotted against different Neuraminidase concentrations (U/ml) (from C. perfringens), measured for 5 min at 25° C.

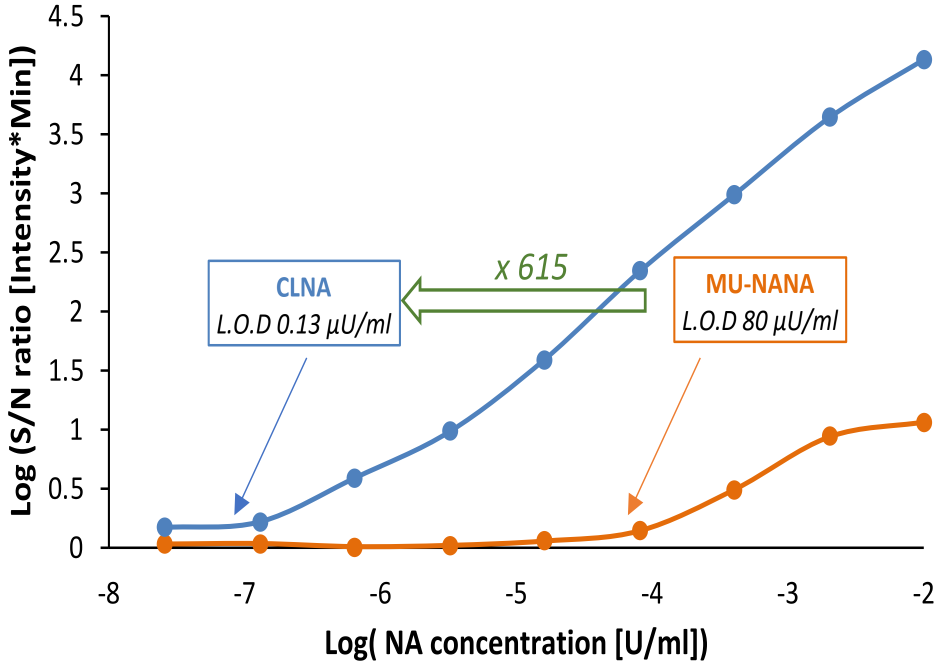

Figure S3. Signal to noise ratio values for **CLNA** [10 uM] and MU-NANA [10uM], plotted against different neuraminidase concentrations [U/ml] (from *C. perfringens*) using logarithmic scales, 1% DMSO in PBS pH 7.4, $CaCl_2$ [4 mM], Measured during 5 minutes at 25°C. L.O.D was defined as blank + 3SD (standard deviation).

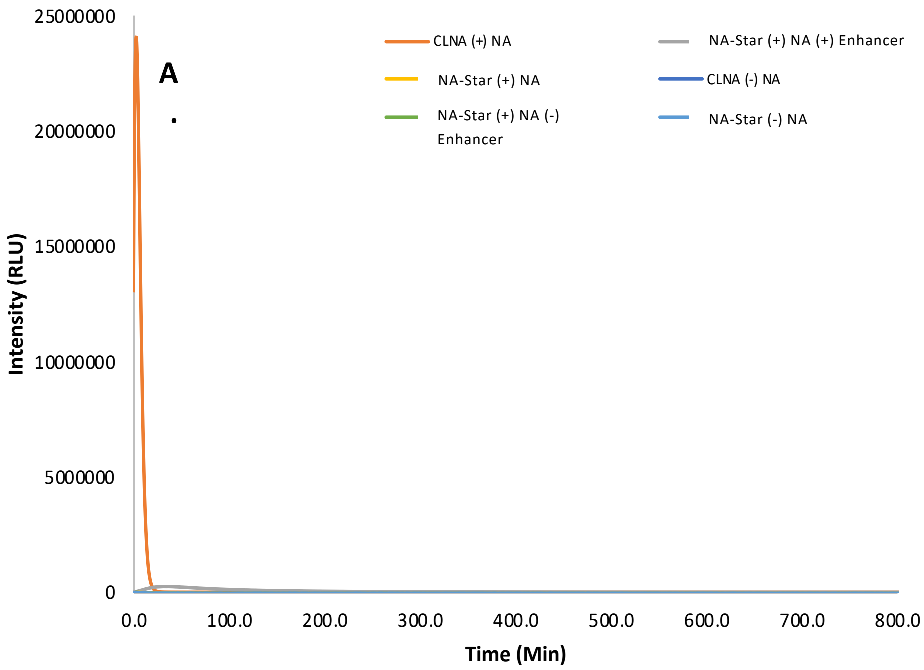

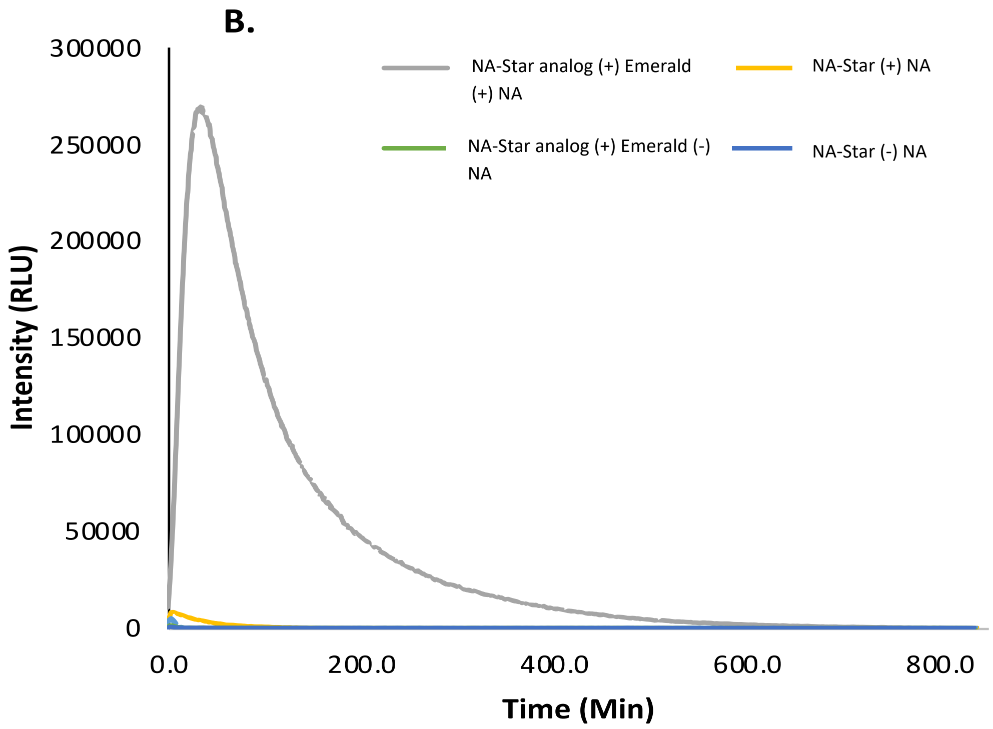

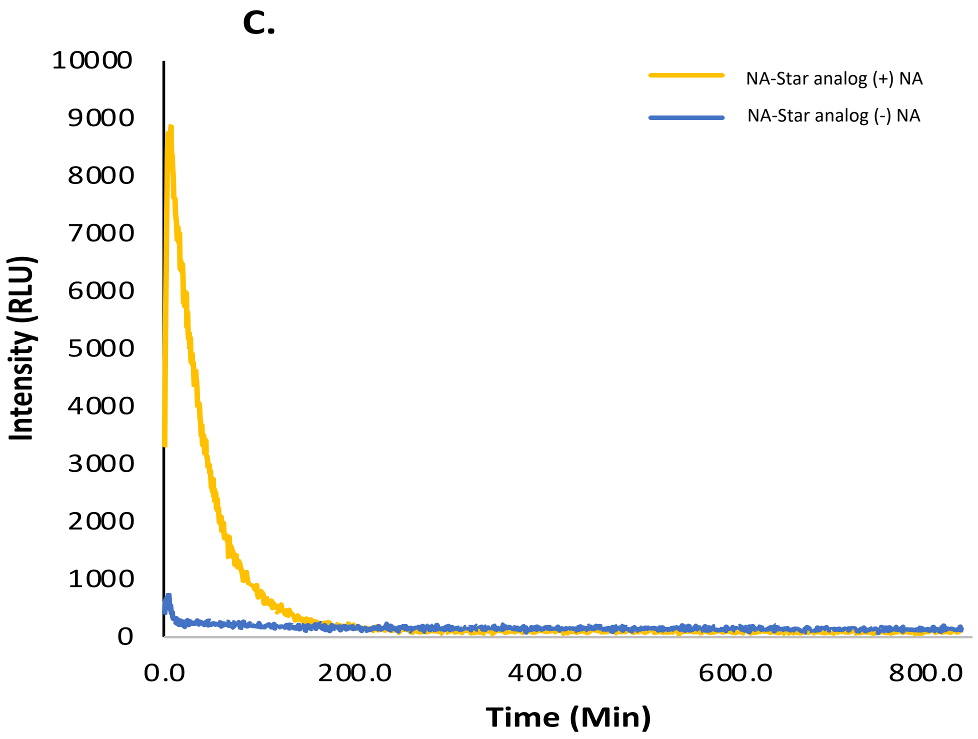

Figure S4. Chemiluminescence kinetic profile of **CLNA** (A) and **NA-Star analog (**B + C) [10µm], in the presence and absence of 10% Emerald-II™ Enhancer, 10% DMSO in PBS pH 7.4, 27°C with and without Neuraminidase (from C. perfringens) [0.1U/ml].

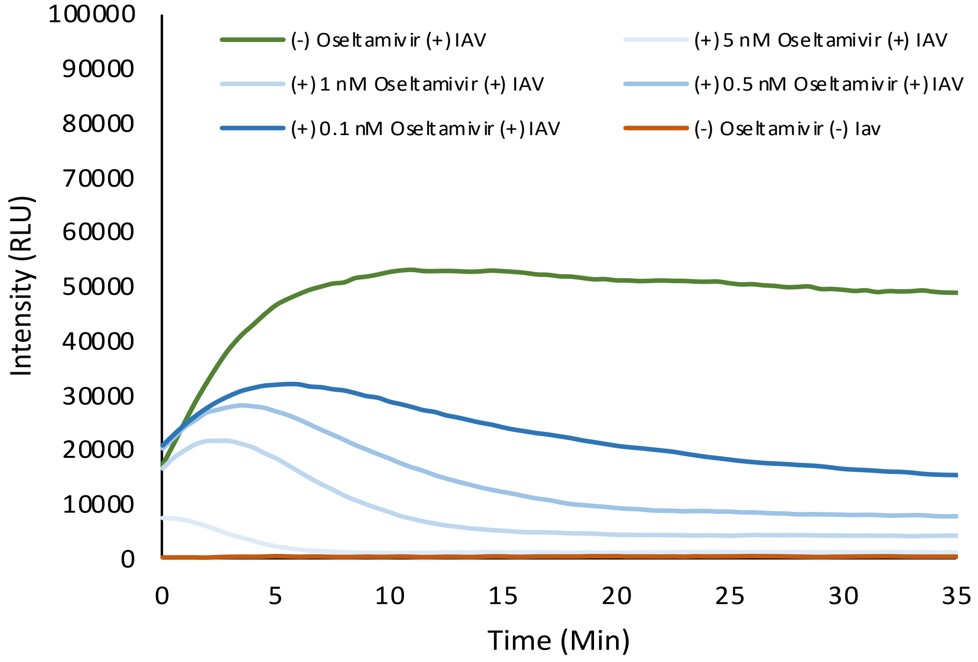

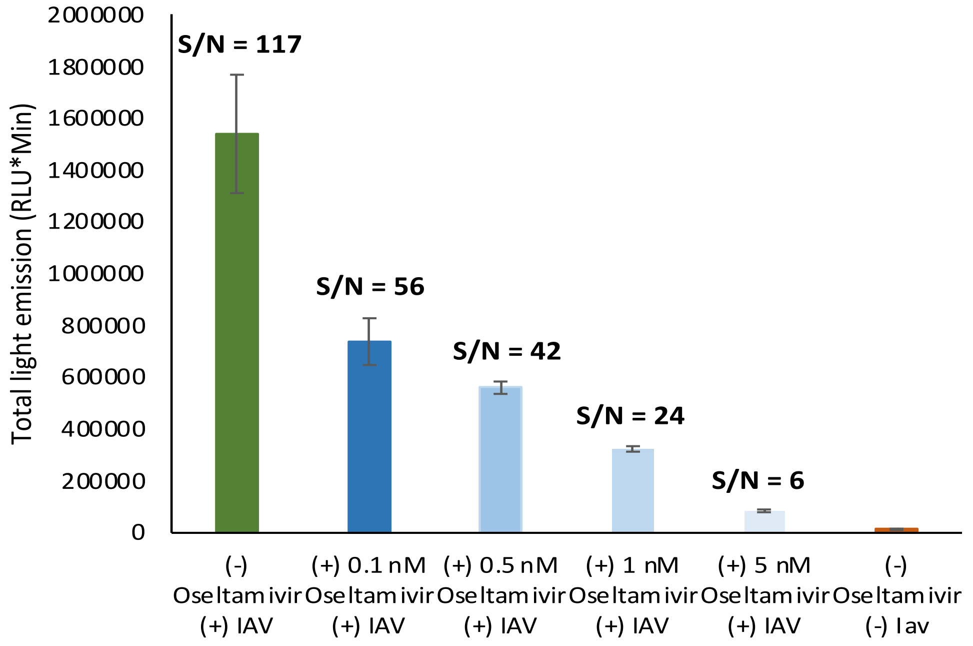

Figure S5. Chemiluminescence kinetic Profile and Signal-to-noise ratio measured after 30 min of **CLNA** [10 µM] in PBS, pH 7.4, 1% DMSO, 37°C in the presence and absence of IAV [10^-2 dilution], and in the presence and absence of Oseltamivir carboxylate [5, 1, 0.5, 0.1 nM]

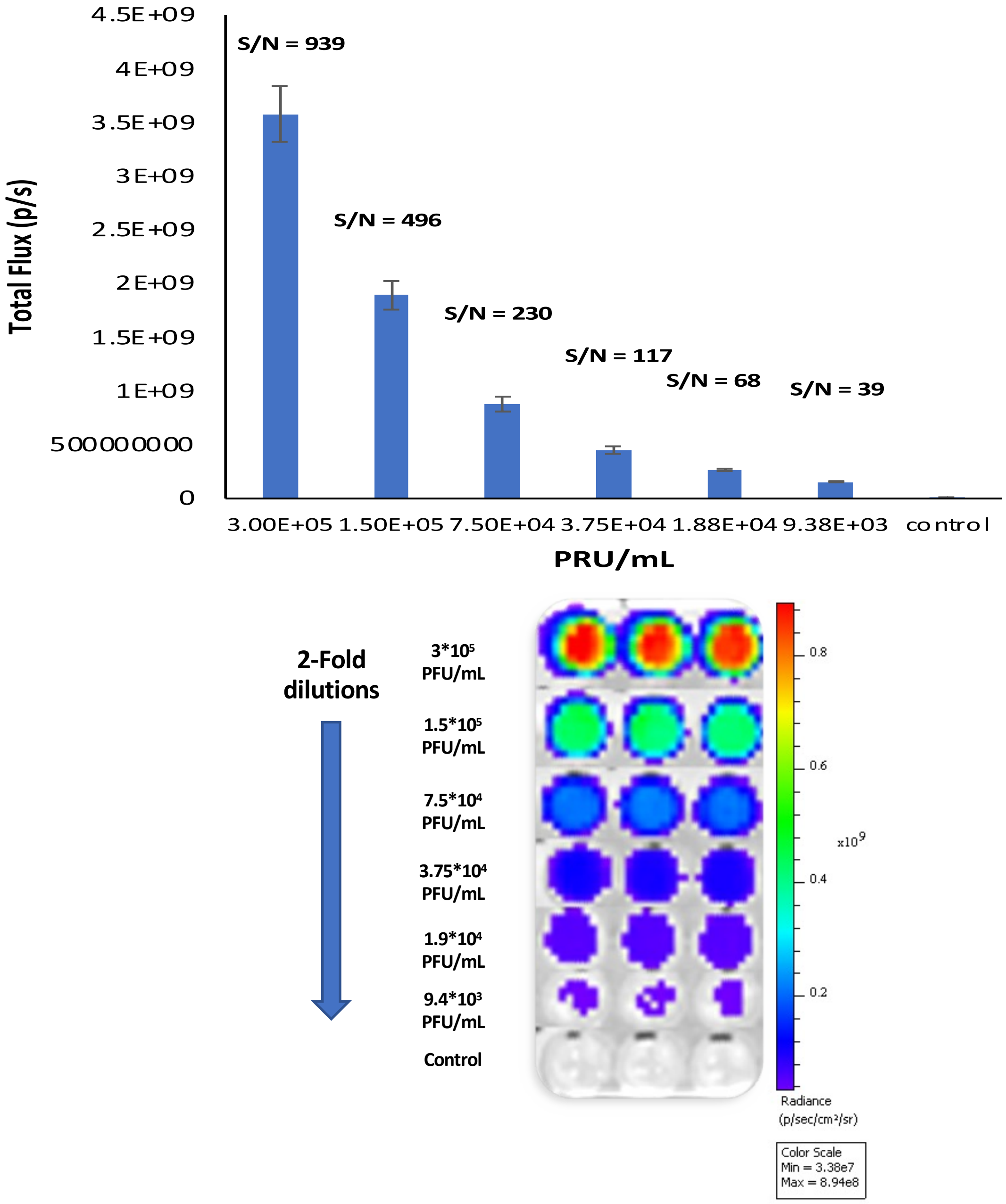

Figure S6. Total flux emitted during 10 second from **CLNA** [10 µm] in PBS, pH 7.4, 1% DMSO, 37°C in the presence of varying IAV infected MDCK cell supernatant [$3*10^5$, $1.5*10^5$,$7.5*10^4$, $3.75*10^4$, $1.9*10^4$, $9.4*10^3$ PFU/mL].

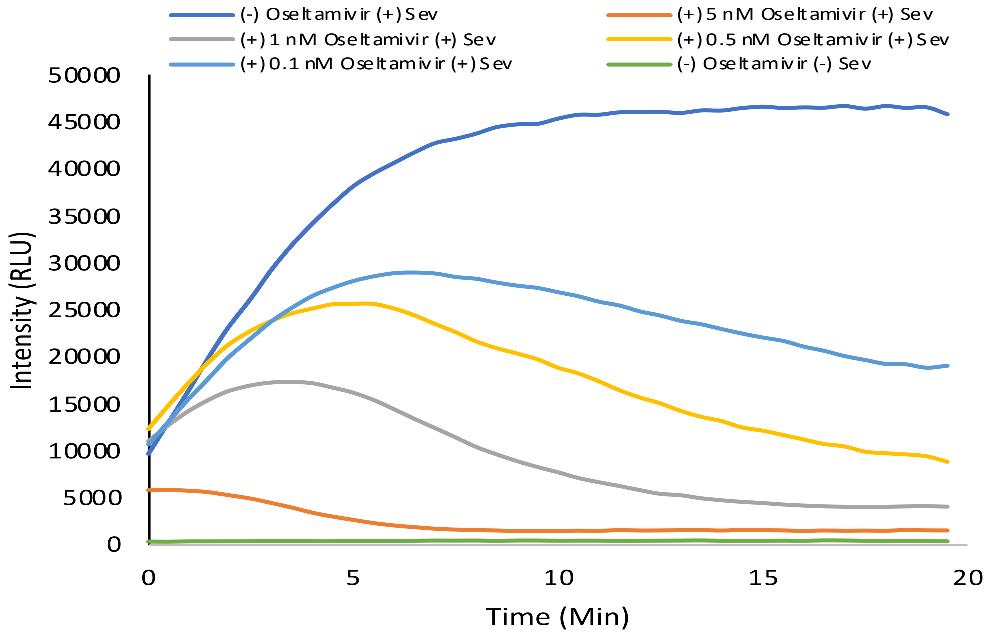

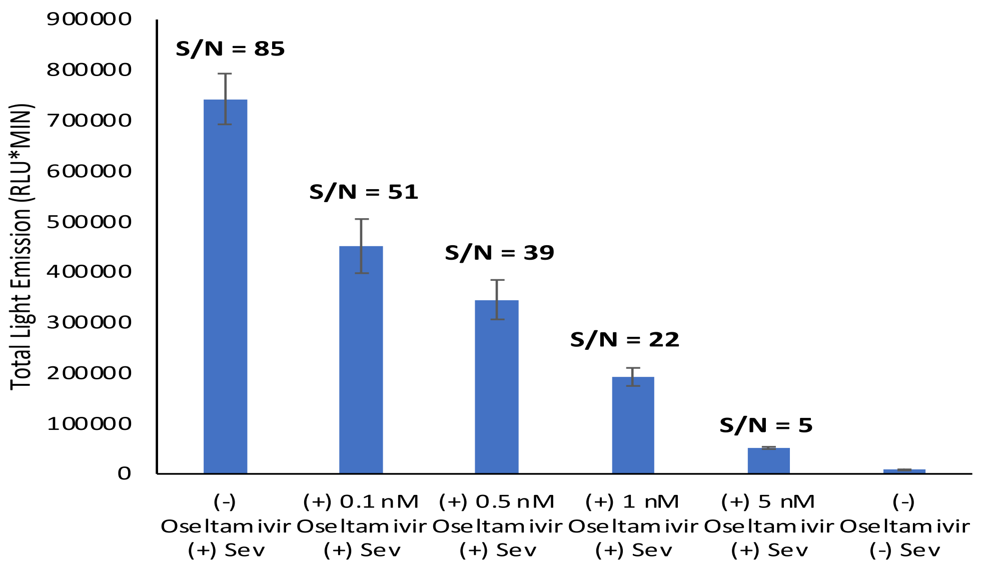

Figure S7. Chemiluminescence kinetic profile and signal-to-noise ratio measured after 20 min of **CLNA** [10 µM] in PBS, pH 7.4, 1% DMSO, 37°C in the presence and absence of Sendai Virus [10^4 PFU/mL] and in the presence and absence of Oseltamivir carboxylate [5, 1, 0.5, 0.1 nM]

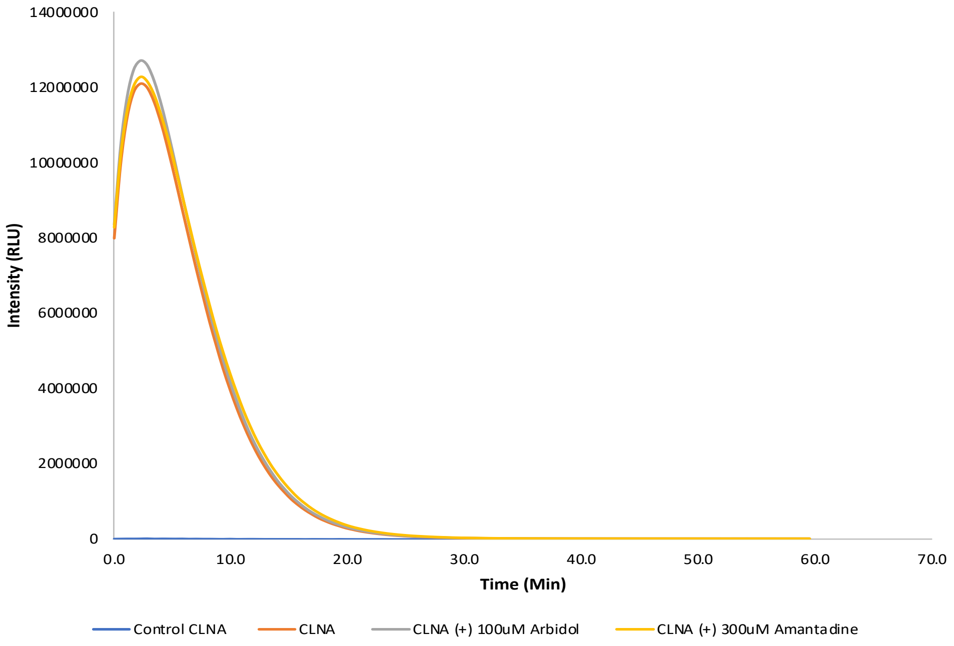

Figure S8. Chemiluminescence kinetic profile **CLNA** [10 µm], in the presence of the highest used concentration of Amantadine and Arbidol [300 µm and 100 µm], 1% DMSO in PBS, in the presence and absence of different *Neuraminidase concentrations* (U/ml) (*from C. perfringens*), measured at 25° C.

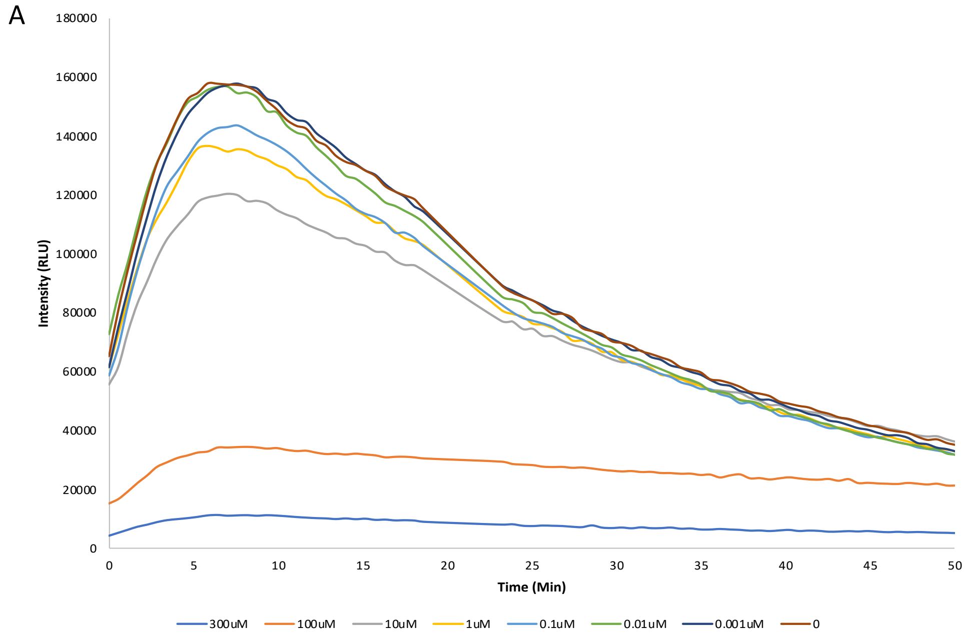

B

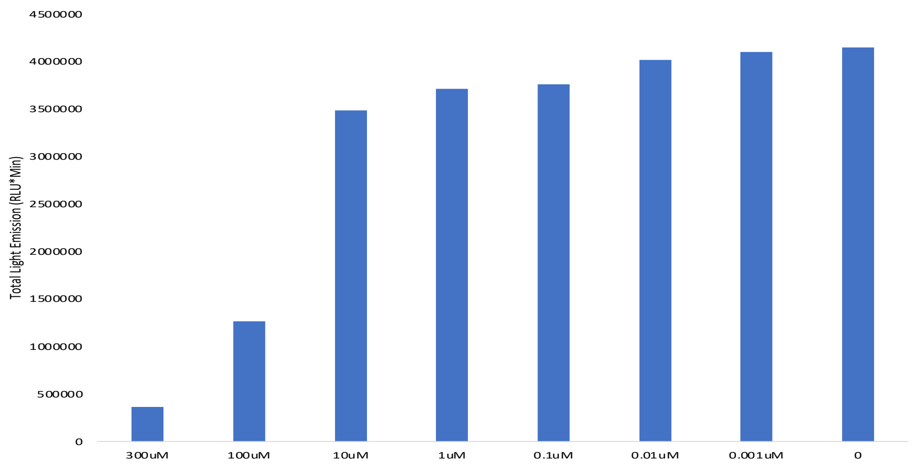

Figure S9. Chemiluminescence kinetic Profile (A) and total light emission measured after 50 min (B) of **CLNA** [10 µM] in PBS, pH 7.4, 1% DMSO, 37°C , in the presence of PR8 (S31N, V27T) infected MDCK cells in the presence of varying concentrations of Amantadine [300 – 0.001 µm].

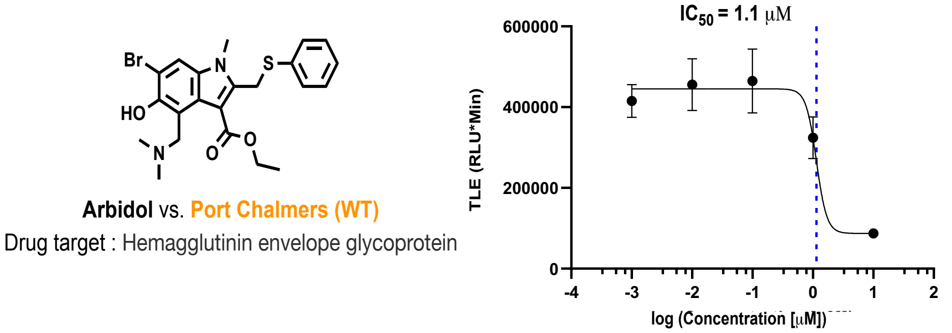

Figure S10. IC_{50} values and sigmoidal fit of Arbidol activity against Port Chalmers. Results were obtained following the general cell assay procedure.

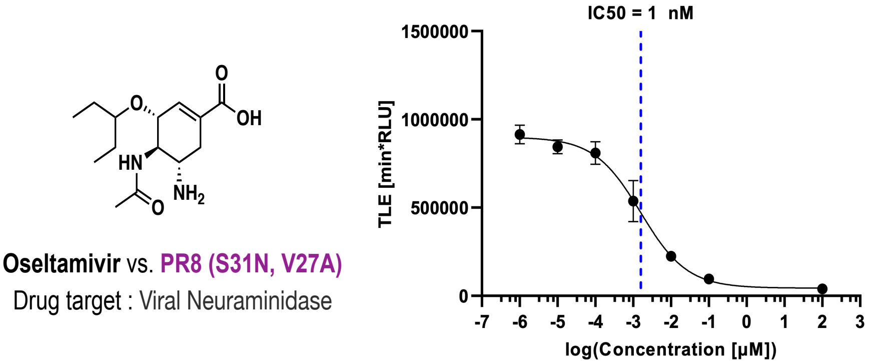

Figure S11. IC_{50} values and sigmoidal fit of Oseltamivir activity against PR8A. Results were obtained following the general cell assay procedure.

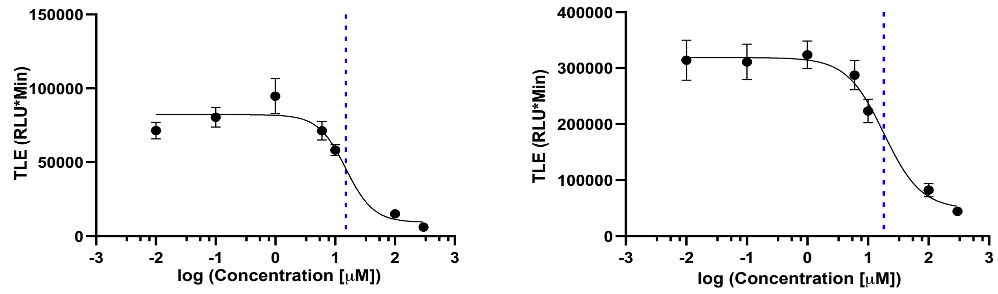

California (S31N, V27): IC_{50} = 15 µM **PR8 (S31N, V27A):** IC_{50} = 18 µM

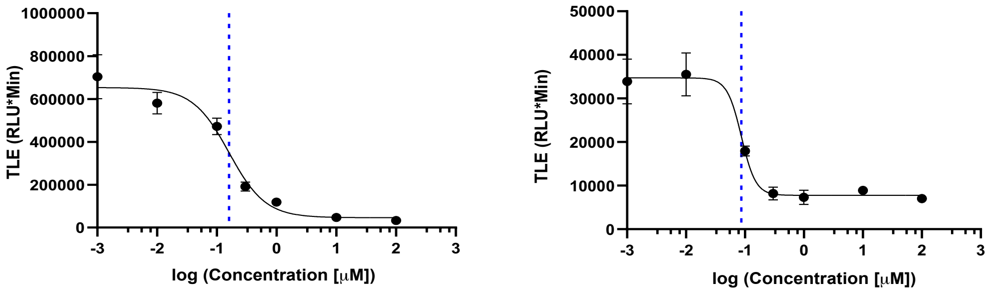

Port Chalmers (WT): IC_{50} = 0.16 µM **Fort Monmouth (WT):** IC_{50} = 0.08 µM

Figure S12. IC_{50} values and sigmoidal fit of Amantadine activity against Port Chalmers, California, PR8A and Fort Monmouth. Results were obtained following the general cell assay procedure.

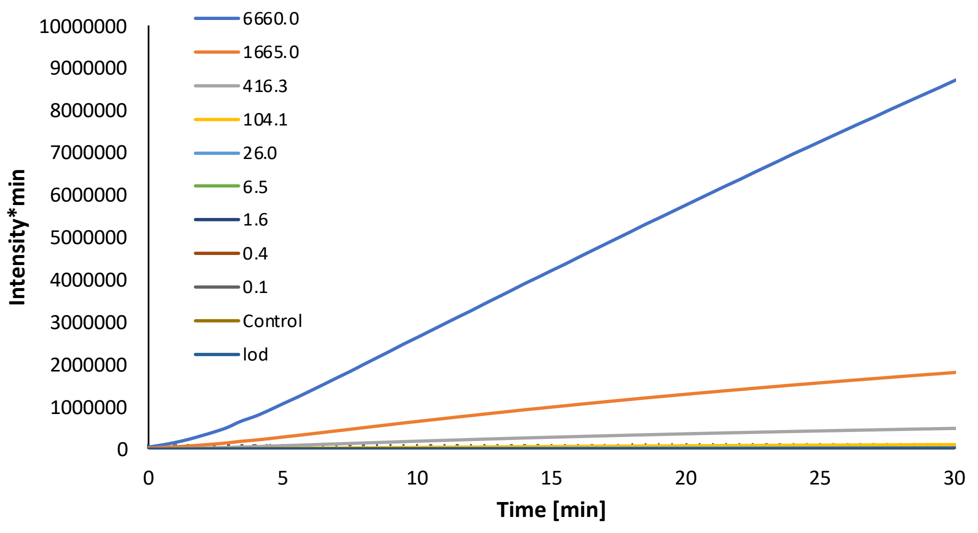

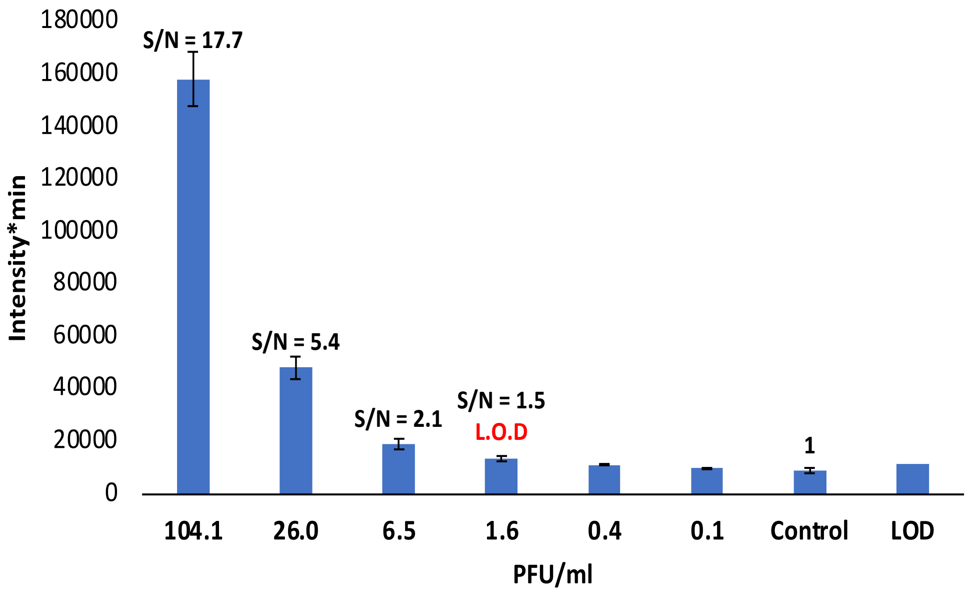

Figure S13. Total light emission plotted against time (up) and Total light emission measured 30 min upon addition of the virus (down) of **CLNA** [10µm], 0.1% DMSO in PBS pH 7.4, 37°C, plotted against varying Influenza virus concentrations [104.1-0.1 PFU/mL).

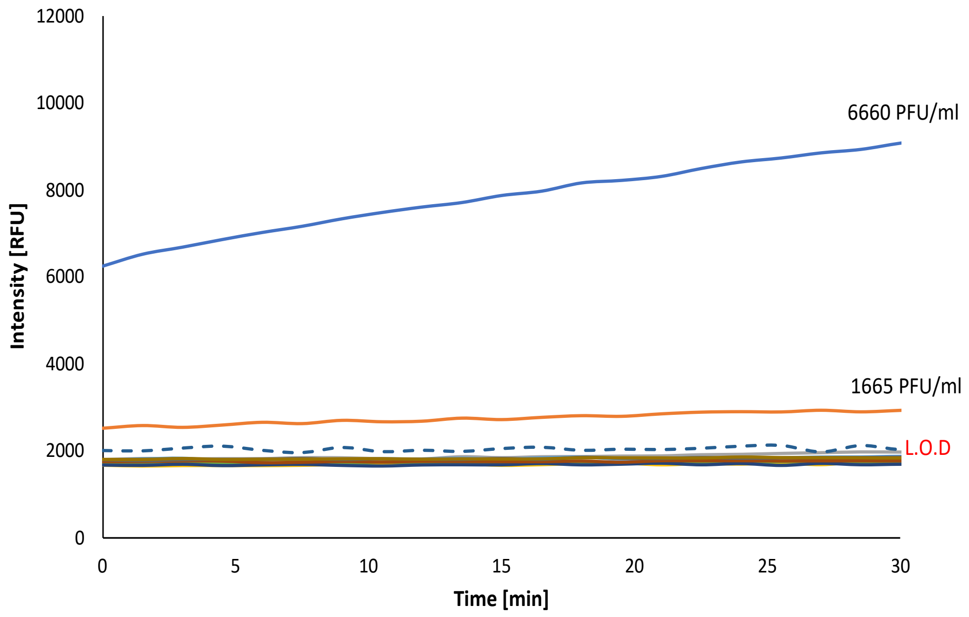

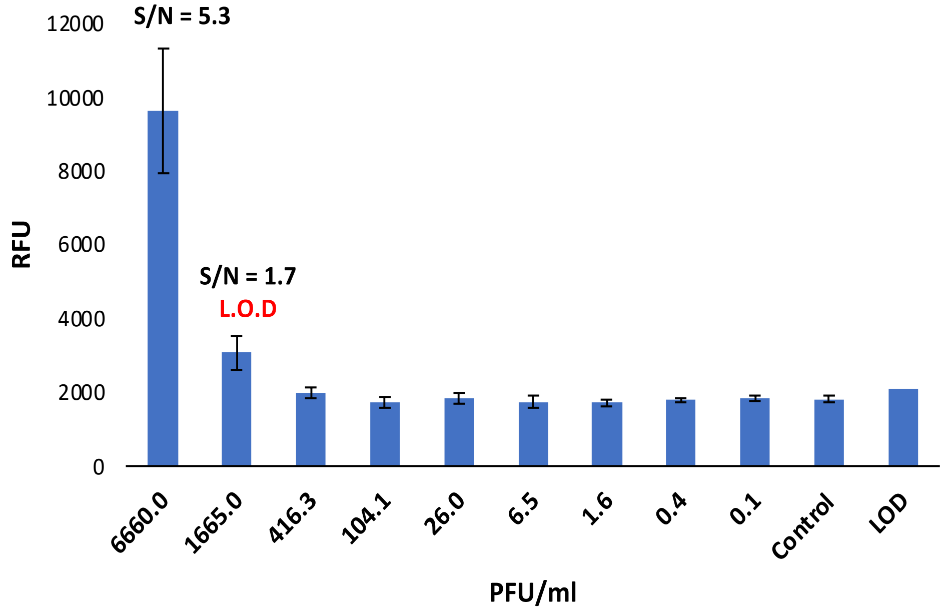

Figure S14. Total light emission plotted against time (up) and Total light emission measured 30 min upon addition of the virus (down) of **MU-NANA** [10µm], 0.1% DMSO in PBS pH 7.4, 37°C, plotted against varying Influenza virus concentrations [104.1-0.1 PFU/mL).

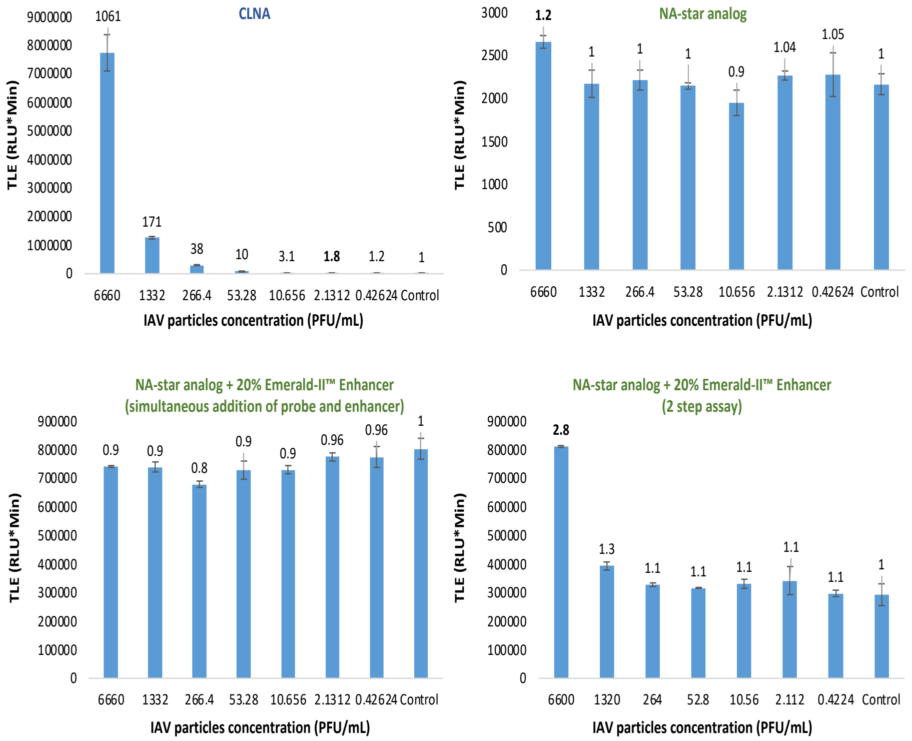

Figure S15. Total light emission measured after 30 min upon addition of the virus to **Neu5Ac NA-Star analog (** [10µm], 0.1% DMSO in PBS pH 7.4, 37°C, plotted against varying Influenza virus concentrations [6660 - 0.46 PFU/mL).

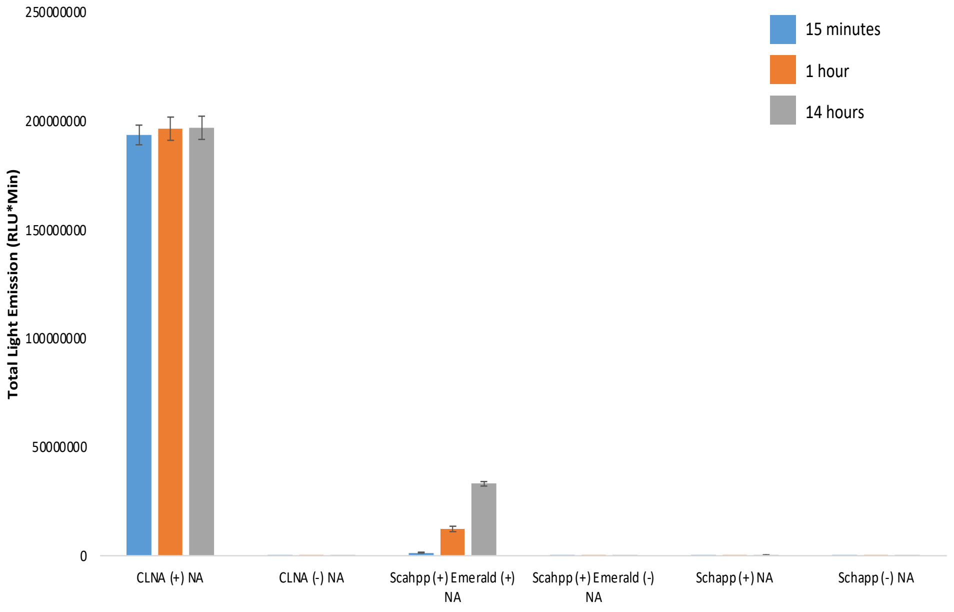

Figure S16. Total light emission of **NA-Star analog (** [10µm], 1% DMSO in PBS 7.4, in the presence and absence of different *Neuraminidase concentrations* (U/ml) (*from C. perfringens*), measured at 25° C after 15 minutes, 1 hour and 14 hours.

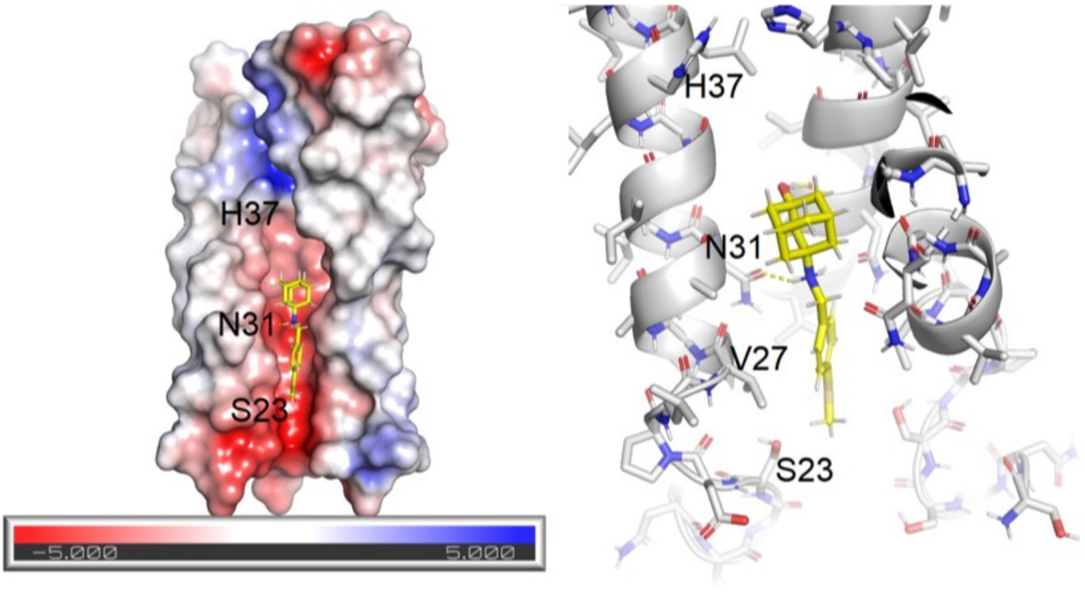

Figure S17. Adaptive Poisson-Bolzmann Solver (APBS) electrostatics surface (With Coulomb scale from -5 [kcal/(mol*e)] (left) in most red to 0 *i.e.,* neutral in white and +5 [kcal/(mol*e), and cartoon and stick representation (right), showing the NMR structure of S31N M2 in the intermediate conformation (PDB ID: 2LY0) with the bound drug (compound **a**) omitted and compound a docked (yellow carbons). One helix from the M2 tetramer was omitted for clarity of presentation. Hydrogen bonds are indicated in dashed yellow.

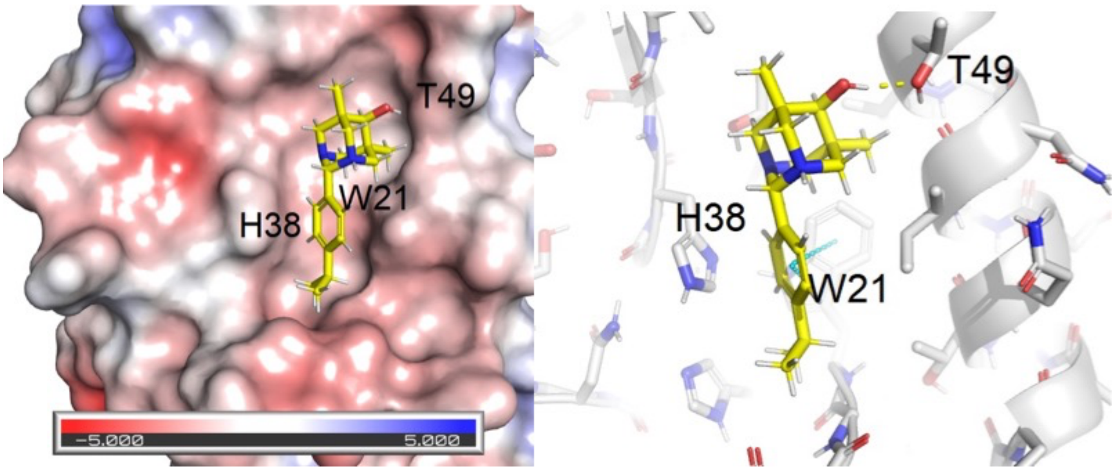

Figure S18. Adaptive Poisson-Bolzmann Solver (APBS) (left) electrostatics surface (With Coulomb scale from -5 [kcal/(mol*e)] in most red to 0 *i.e.,* neutral in white and +5 [kcal/(mol*e)] in most blue) and cartoon and stick representation (right), showing the X-ray crystal structure of PR8 HA(PDB ID: 6WCR) with the bound antiviral drug (compound **a**) omitted and compound b docked (yellow carbons).Hydrogen bonds are indicated in dashed yellow and T-shaped π-stacking interactions in dashed cyan.

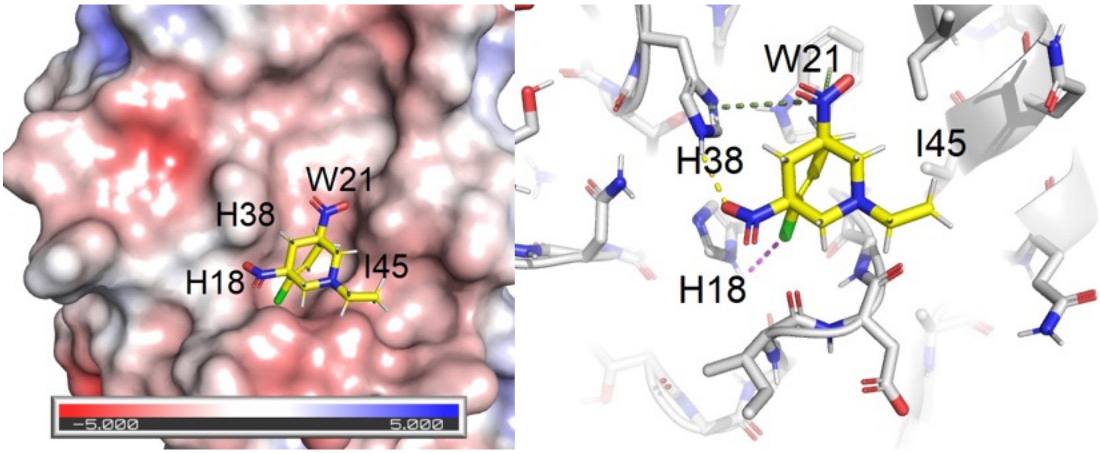

Figure S19. Adaptive Poisson-Bolzmann Solver (APBS) (left) electrostatics surface (With Coulomb scale from -5 [kcal/(mol*e)] in most red to 0 *i.e.,* neutral in white and +5 [kcal/(mol*e)] in most blue) and cartoon and stick representation (right), showing the X-ray crystal structure of PR8 HA (PDB ID: 6WCR) with the bound antiviral drug omitted and compound c docked (yellow carbons). Hydrogen bonds are indicated in dashed yellow, halogen bonds in dashed magenta and π-cation interactions in dashed dark green.

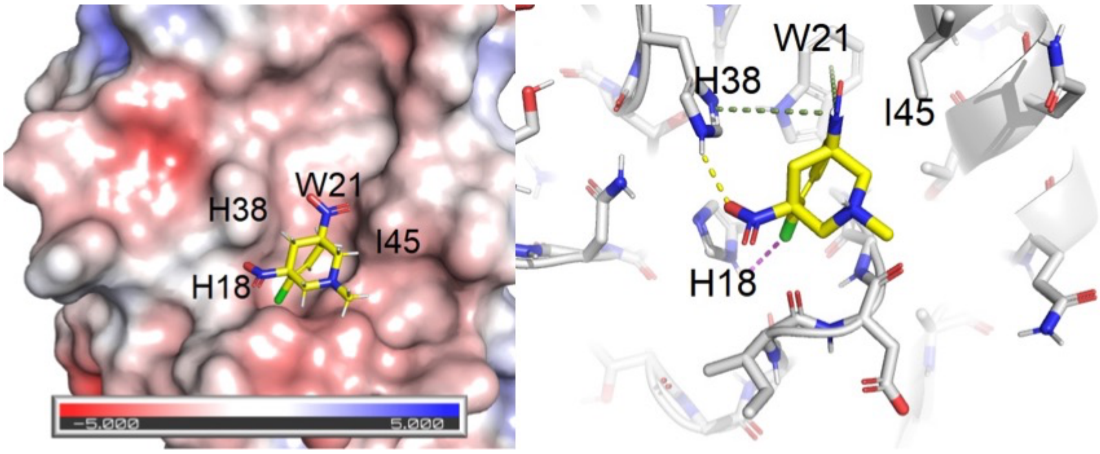

Figure S20. Adaptive Poisson-Bolzmann Solver (APBS) (left) electrostatics surface (With Coulomb scale from -5 [kcal/(mol*e)] in most red to 0 *i.e.,* neutral in white and +5 [kcal/(mol*e)] in most blue) and cartoon and stick representation (right), showing the X-ray crystal structure of PR8 HA (PDB ID: 6WCR) with the bound antiviral drug omitted and compound i docked (yellow carbons). Hydrogen bonds are indicated in dashed yellow, halogen bonds in dashed magenta and π-cation in dashed dark green.

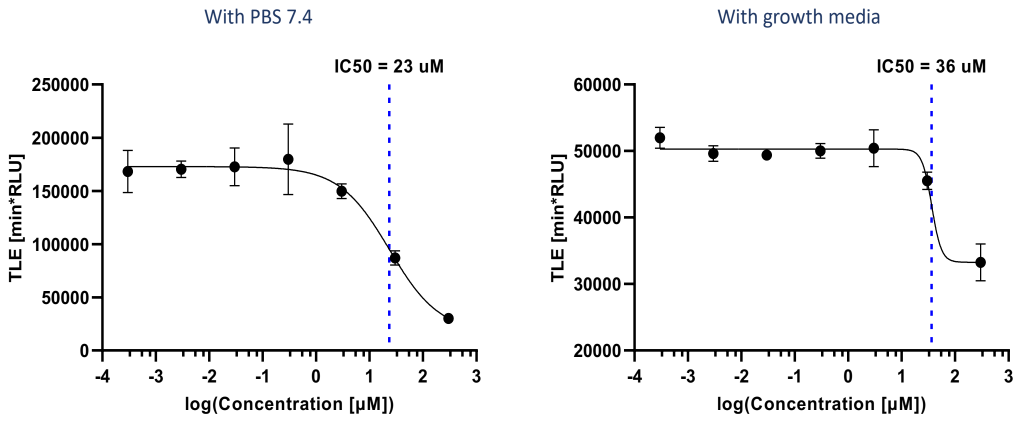

Figure S21. IC_{50} values and sigmoidal fit of Amantadine activity against PR8T. Evaluation of the effect of growth media aspiration on the acquired IC50 values. The left cell-based assay was obtained following the general cell assay procedure, while the right cell-based assay was obtained following the general cell assay procedure with one exception; the media was replaced with a Phenol red-free MEM media (Gibco™), 2% trypsin supplemented with 2% FCS and 1.2 μg/ml trypsin T0303. *Phenol red is known to affect the light emission measured; therefore, phenol red-free media is necessary for the measurements.[1]

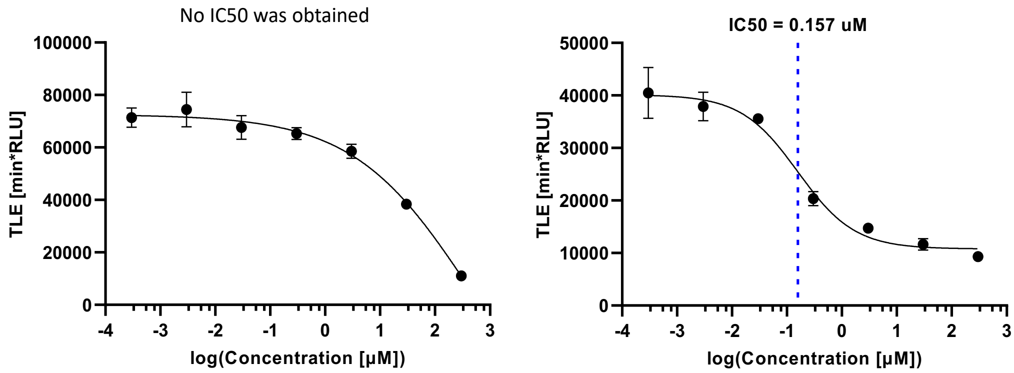

Figure S22. IC_{50} values and sigmoidal fit of Amantadine activity against PR8T (left) and Fort Monmouth (right). Results were obtained after incubation of cells with **NA-Star analog** [10μM] for 30 minutes and the addition of 20% Emerald-II™ Enhancer. Total light emission was measured for 13 minutes upon the addition of the enhancer at 37C.

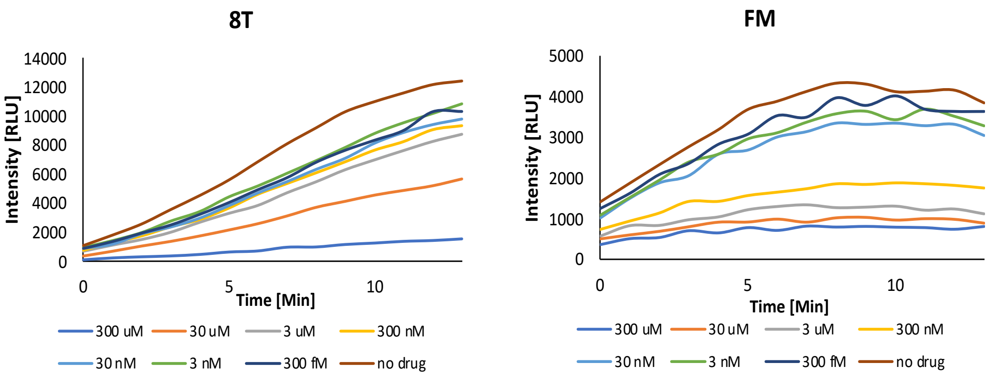

Figure S23. Chemiluminescence kinetic profile with various Amantadine concentrations against PR8T (left) and Fort Monmouth (right). Results were obtained after incubation of cells with **NA-Star analog** [10µM] for 30 minutes and the addition of 20% Emerald-II™ Enhancer. Total light emission was measured for 13 minutes upon the addition of the enhancer at 37C.

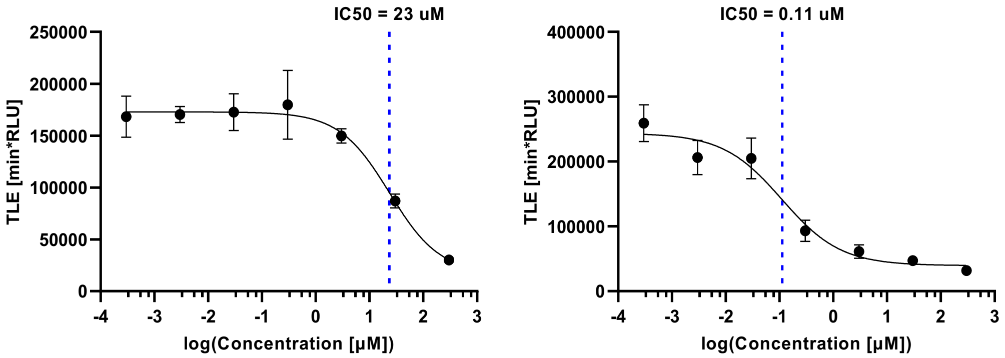

Figure S24. IC_{50} values and sigmoidal fit of Amantadine activity against PR8T (left) and Fort Monmouth (right) as measured with **CLNA** [10µM]. Results were obtained following the general cell assay procedure, for the purpose of comparing **CLNA** and **NA-Star analog** ability to detect Amantadine activity in a cell-based assay.

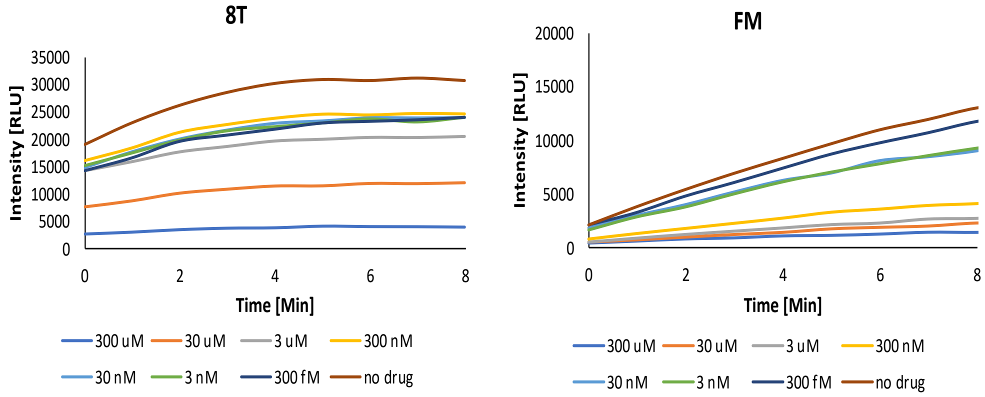

Figure S25. Chemiluminescence kinetic profile with various Amantadine concentrations against PR8T (left) and Fort Monmouth (right). Results were obtained with **CLNA** [10µM] and total light emission was measured for 8 minutes at 37C.

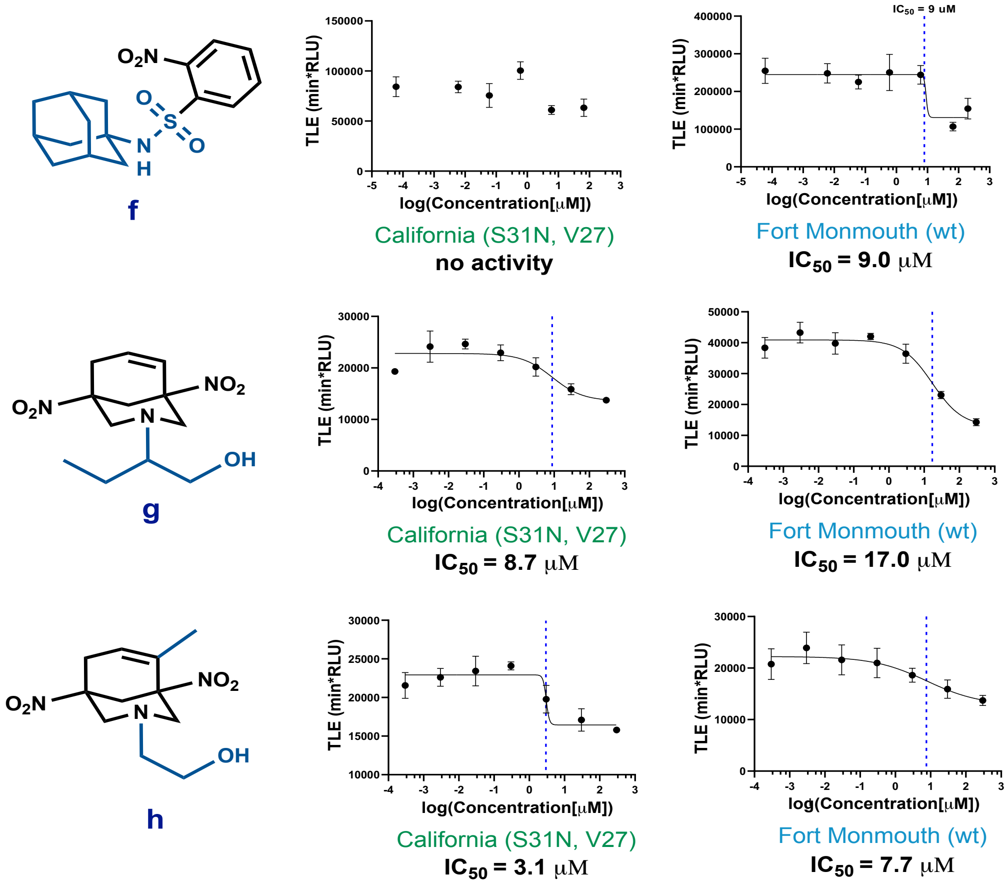

Figure S26. IC_{50} values and sigmoidal fits of compound **f-h** activity against California and Fort Monmouth. Results were obtained following the general cell assay procedure.

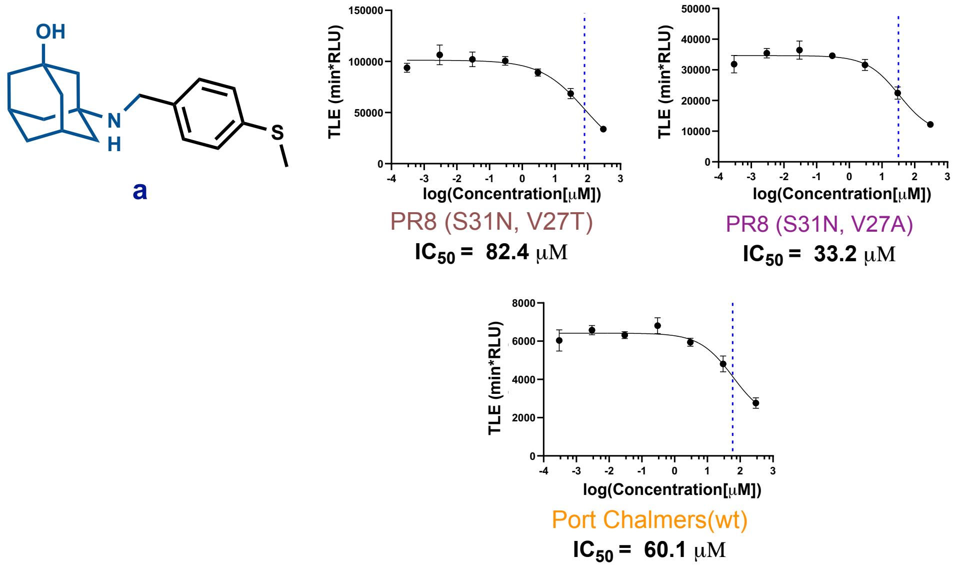

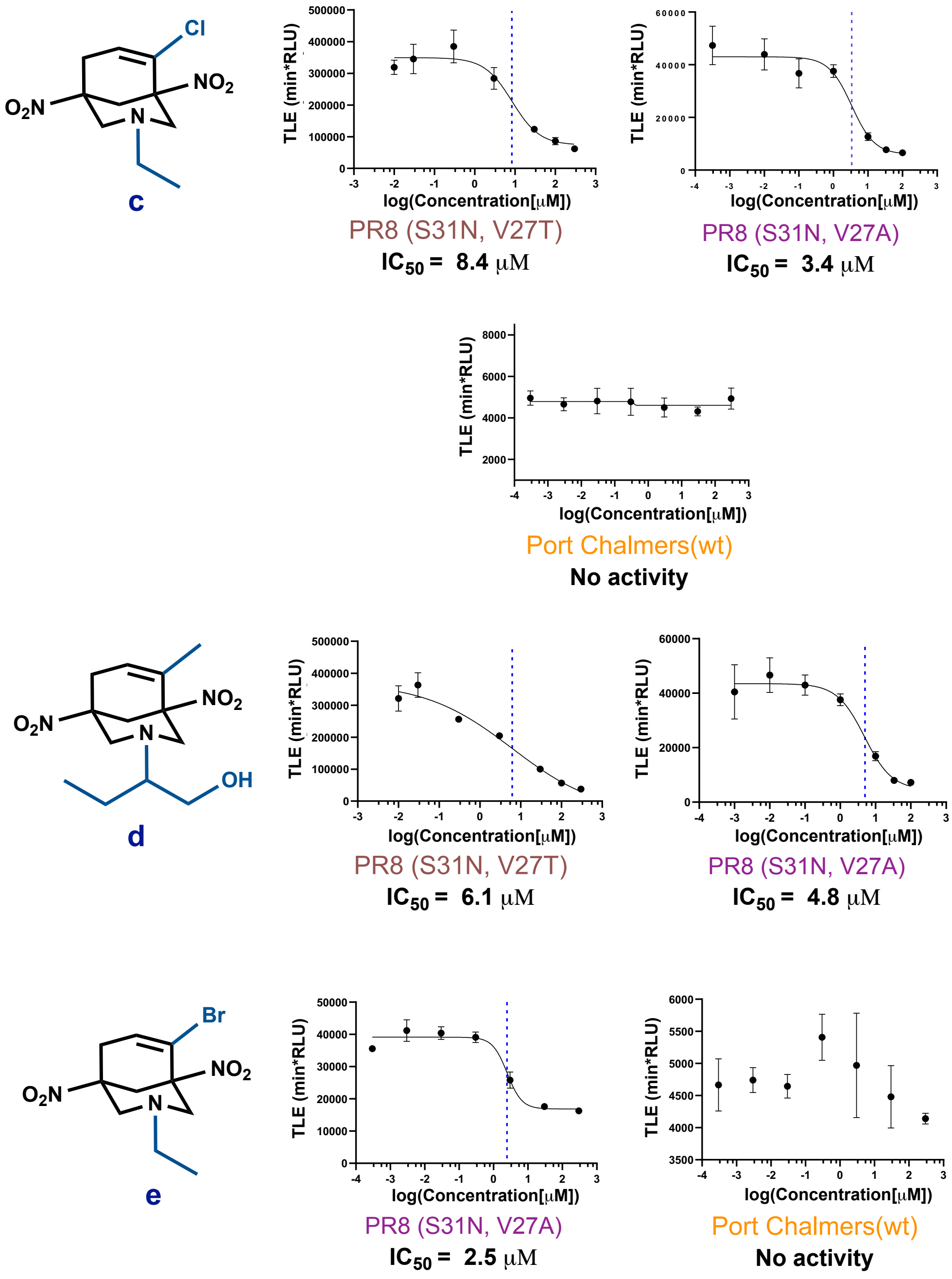

Figure S27. IC_{50} values and sigmoidal fit of Compound **c-e** activity against Port Chalmers, PR8A, and PR8T. Results were obtained following the general cell assay procedure.

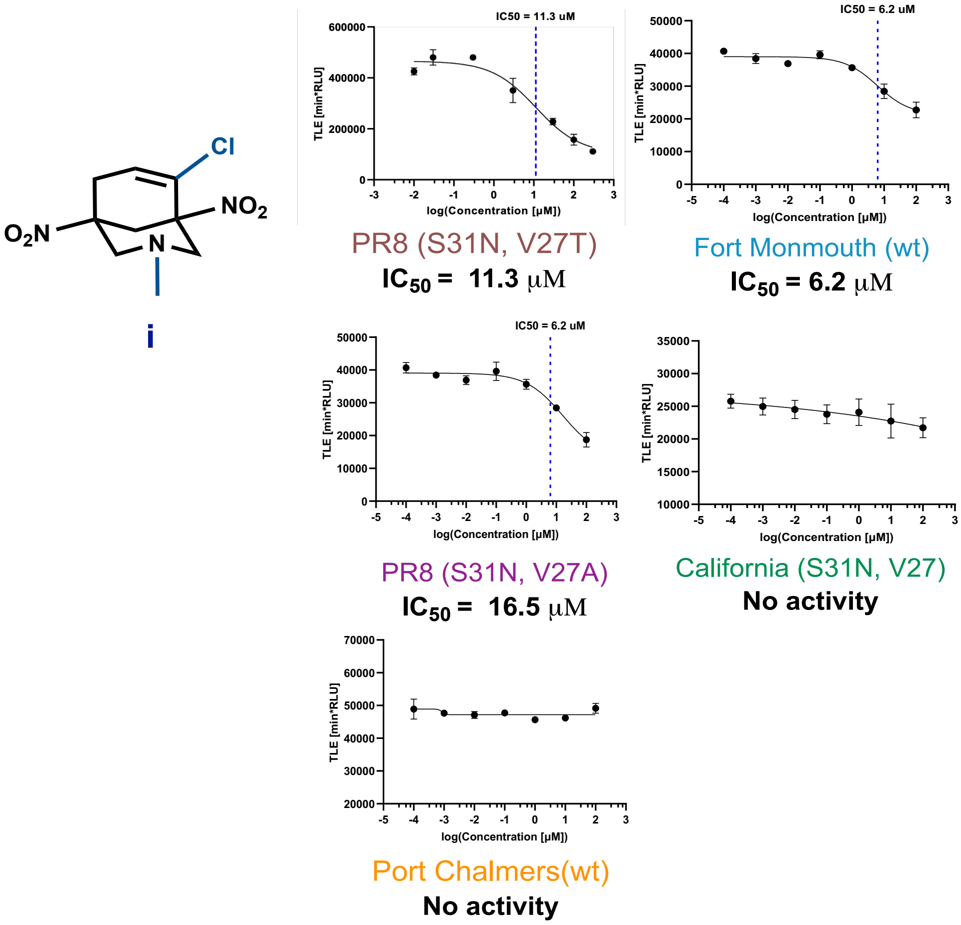

Figure S28. IC_{50} values and sigmoidal fit of Compound **i** activity against Port Chalmers, PR8A, Fort Monmouth, California, and PR8T. Results were obtained following the general cell assay procedure.

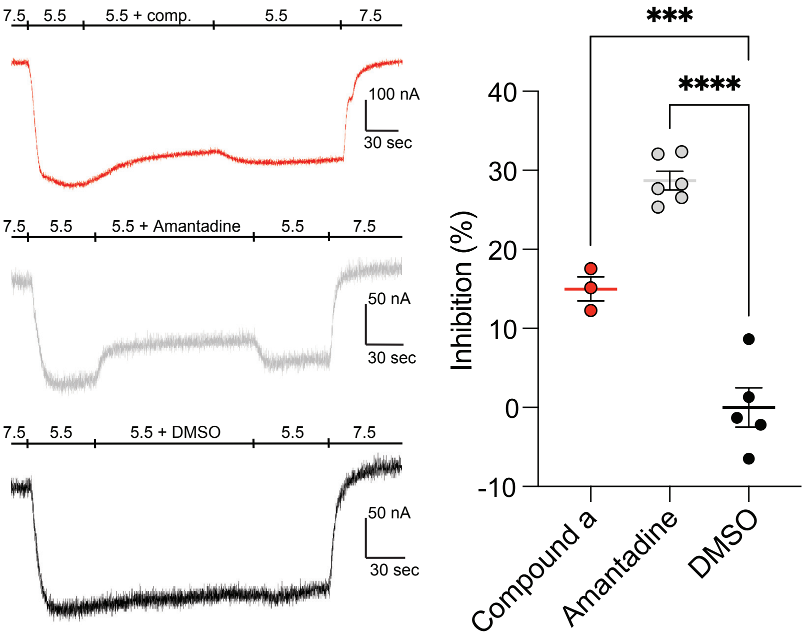

Figure S29. Representative current traces of M2 channel (left), exposed to 100 µM compound **a** (upper), 100 µM Amantadine (middle), or to DMSO (lower). The oocytes were held at a constant voltage of -20 mV. The experimental scheme (pH level and compound application) is indicated by the bars above the traces. Mean percentage inhibition analysis (right) of the small-molecule inhibitors tested, evaluated by a current amplitude ratio at 60 sec and 180 sec. The DMSO mean ratio was subtracted from all groups. (*** $p < 0.001$, **** $p < 0.0001$; one-way ANOVA; n=3 (compound **a**), n=6 (Amantadine), n=5 (DMSO).

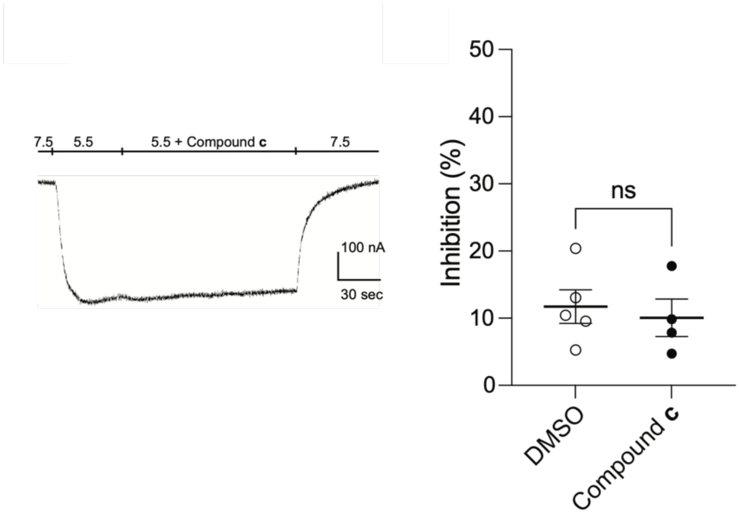

Figure S30. Representative current traces of M2 channel (left), exposed to 100 µM compound **c**. The oocytes were held at a constant voltage of -20 mV. The experimental scheme (pH level and compound **c** application) is indicated by the bar above the traces. Mean percentage inhibition analysis (right) of the small-molecule inhibitors tested, evaluated by a current amplitude ratio at 60 sec and 180 sec. The DMSO mean ratio was subtracted from all groups. (ns-not significant, unpaired t-test; n=4 (compound **c**), n=5 (DMSO).

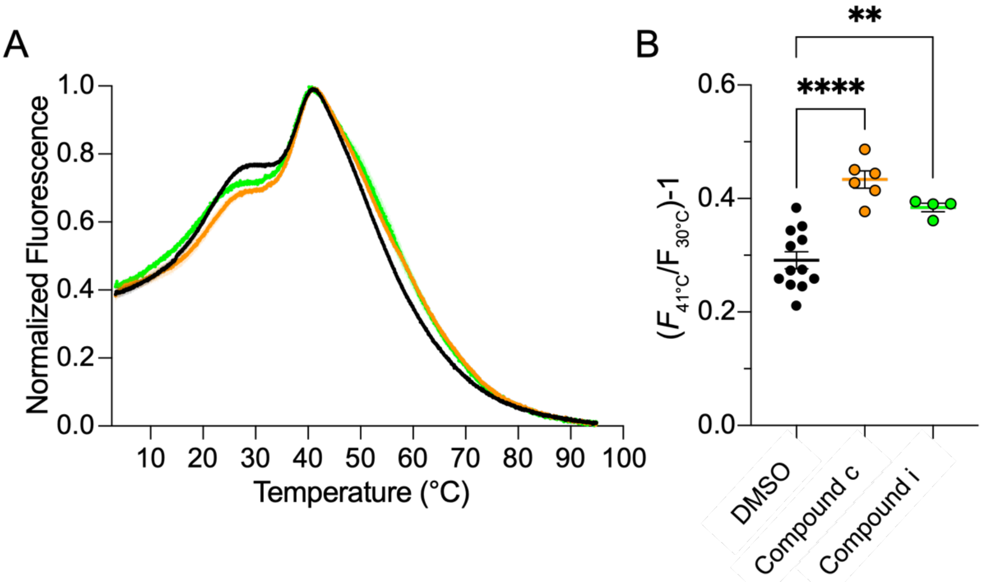

Figure S31. Normalized protein denaturation curves of hemagglutinin (left), measured using Sypro orange fluorescence in the presence of 100 µM compound **c** (orange) and compound **i** (green) or DMSO (black). Fluorescence ratio analysis (right). The change of mean ratio between 41°C and 30 °C fluorescence was used to indicate small-molecule interaction with the hemagglutinin. (**p < 0.01, **** p < 0.0001, one way ANOVA; n=12 (DMSO), n=6 (compound c), n=4 (compound **i**)).

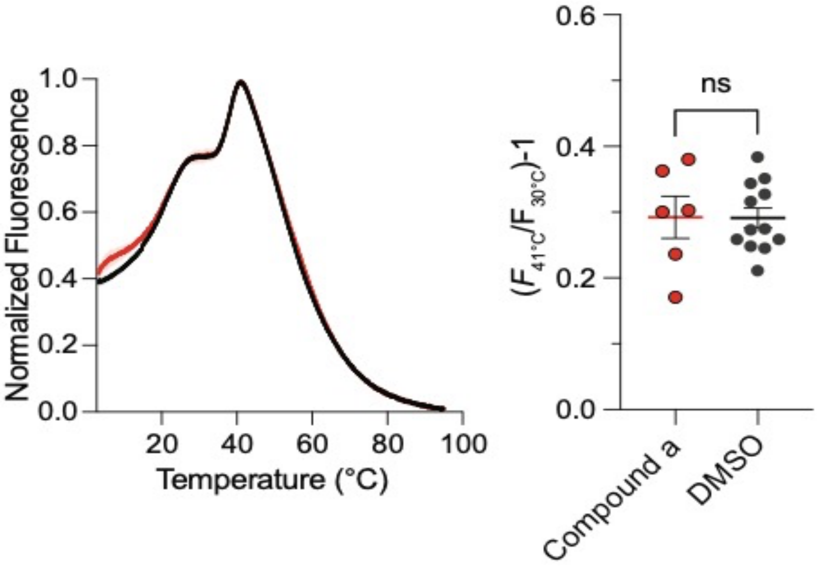

Figure S32. Normalized protein denaturation curves of hemagglutinin (left) measured using Sypro orange fluorescence in the presence of 100 µM compound **a** (red) or DMSO (black). Fluorescence ratio analysis (right). The change of mean ratio between 41°C and 30 °C fluorescence was used to indicate small-molecule interaction with the hemagglutinin. ns-not significant, unpaired t-test; n=12 (DMSO)).

3. NMR spectra

^{1}H-NMR spectra of compound **1**

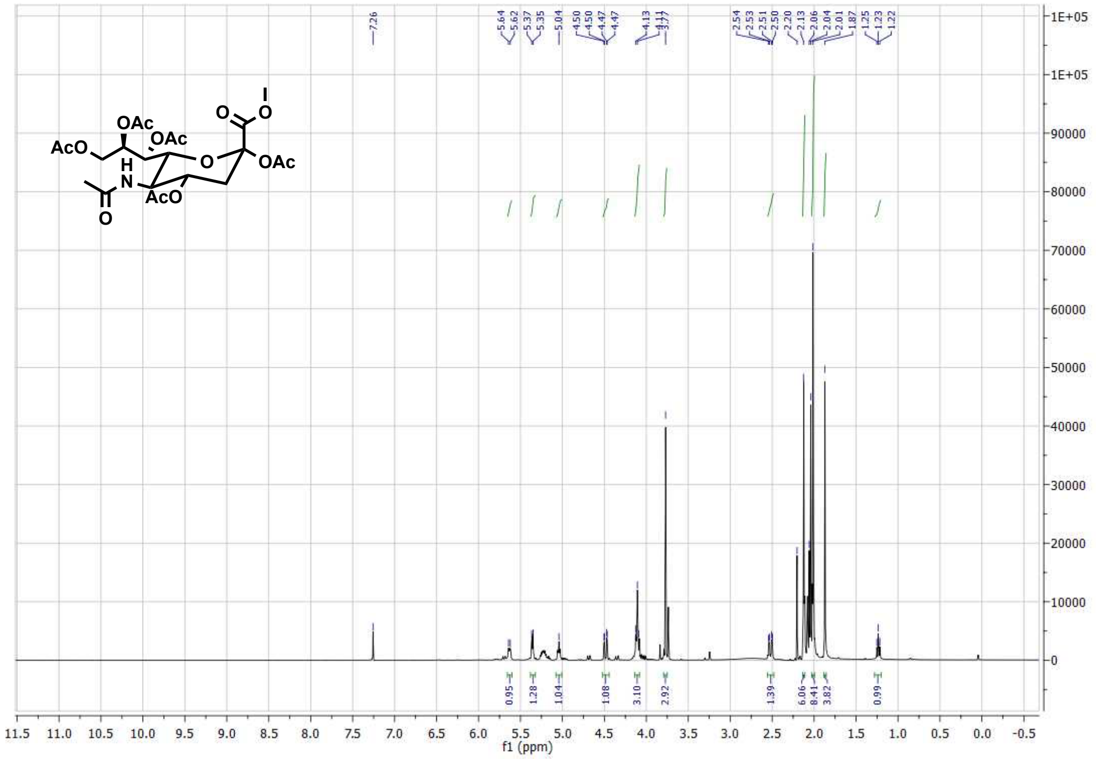

^{13}C-NMR spectra of compound **1**

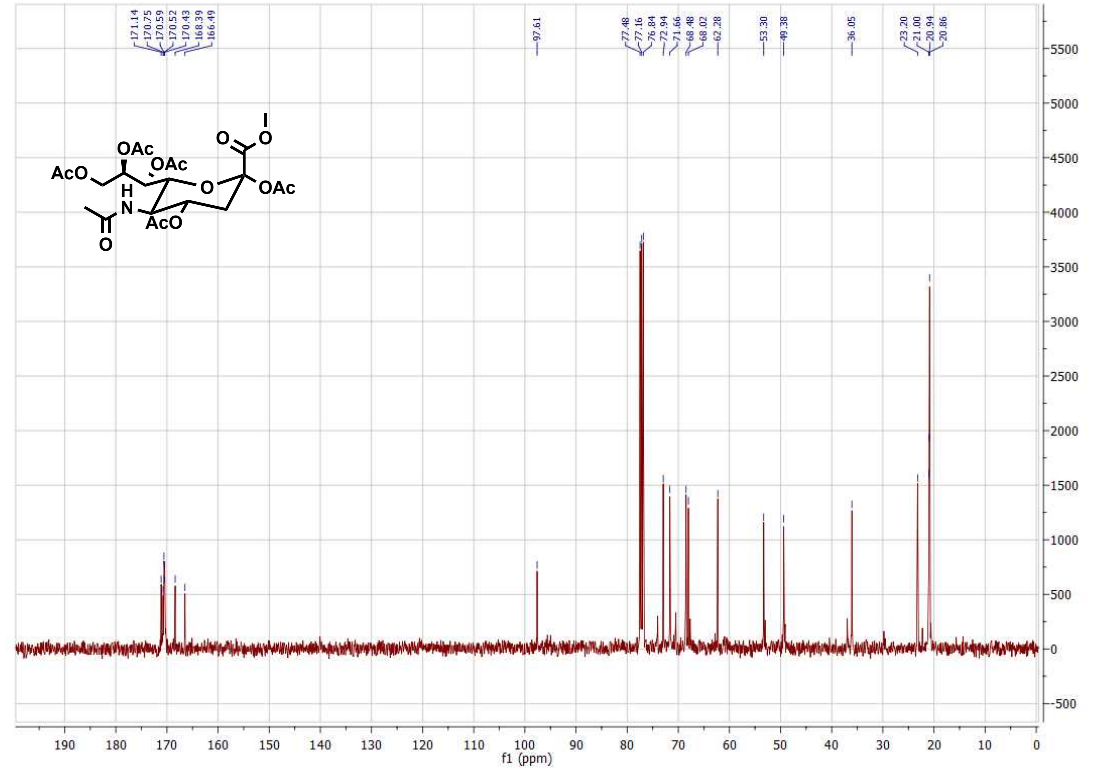

^{1}H-NMR spectra of compound **2**

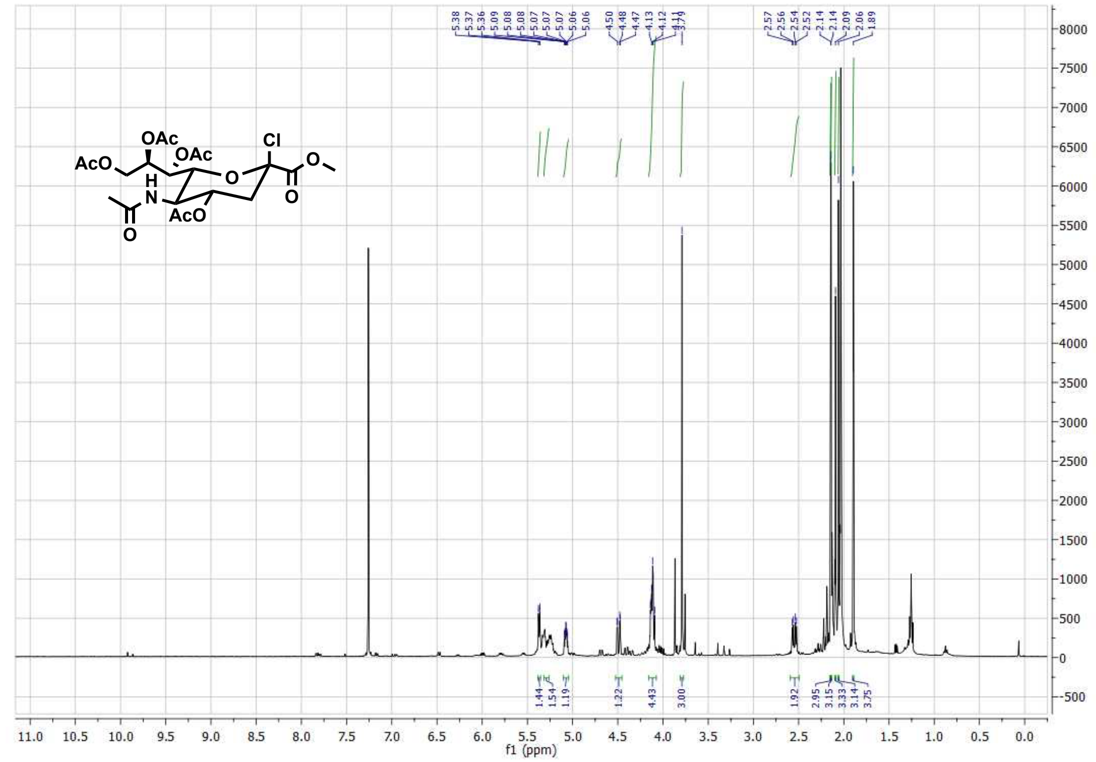

^{13}C-NMR spectra of compound **2**

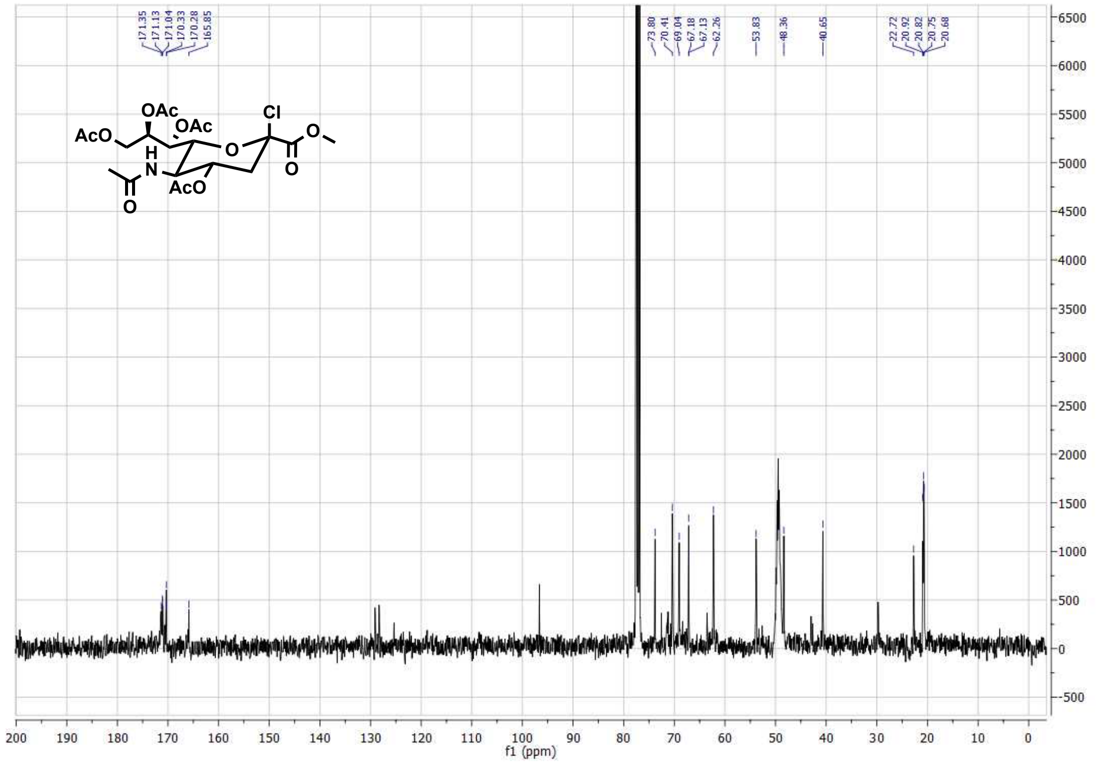

^{1}H-NMR spectra of compound **3**

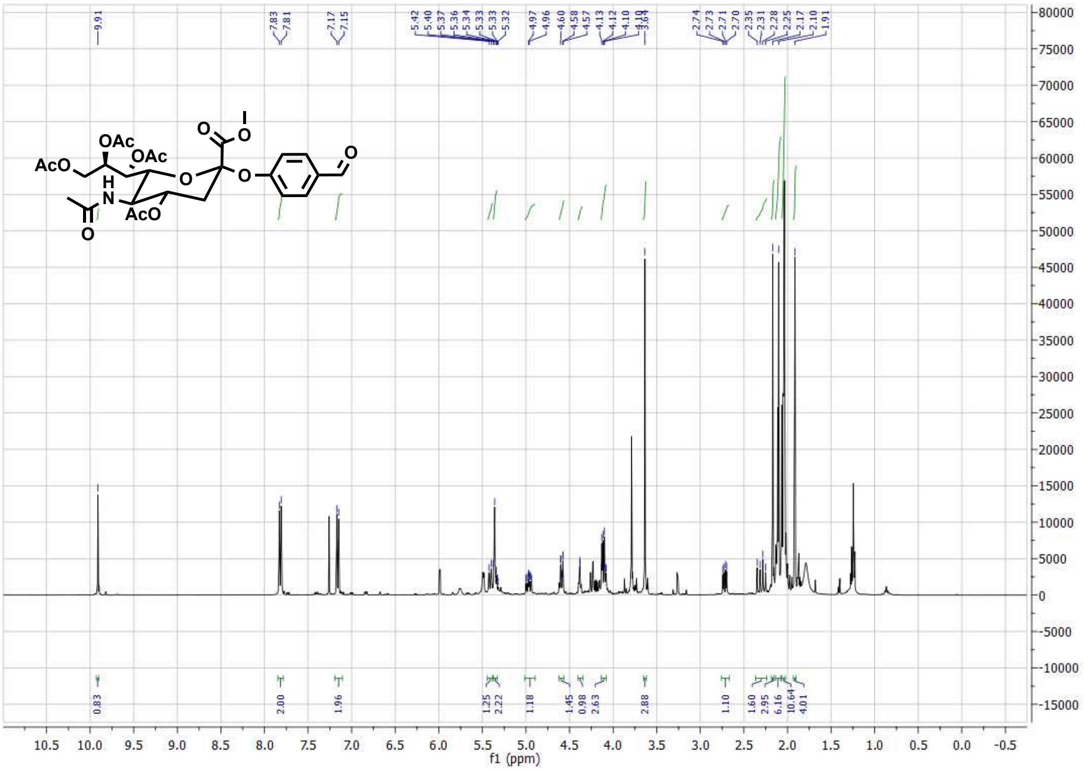

^{13}C-NMR spectra of compound **3**

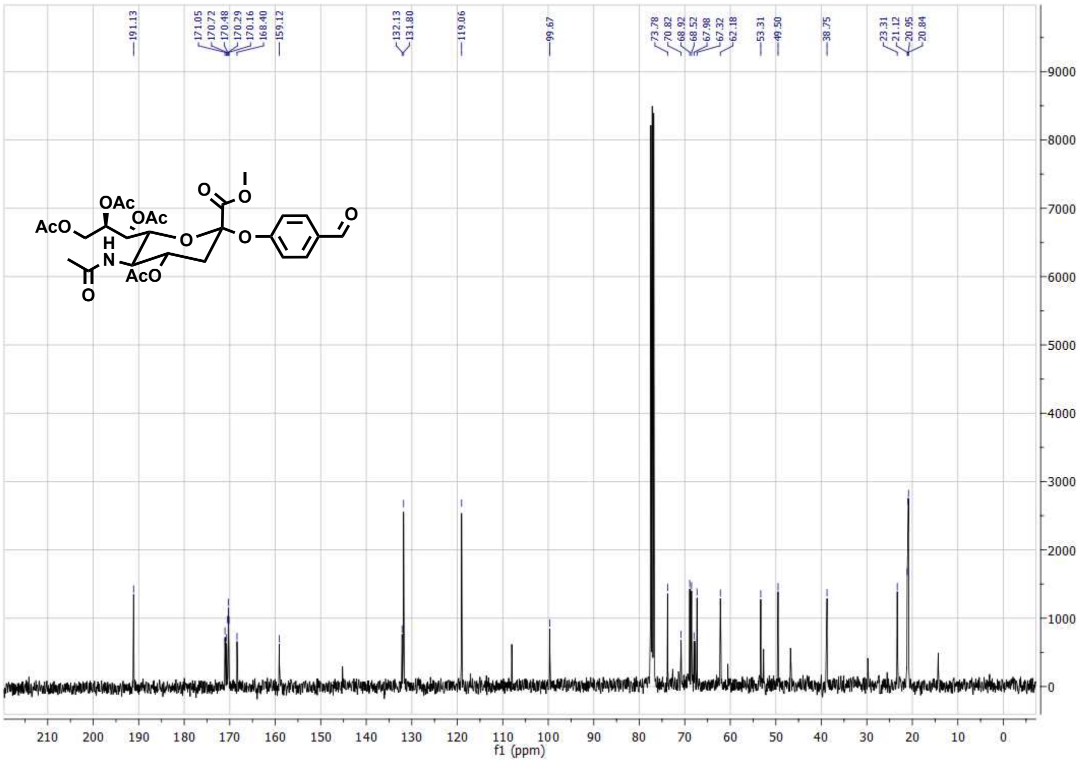

^{1}H-NMR spectra of compound **4**

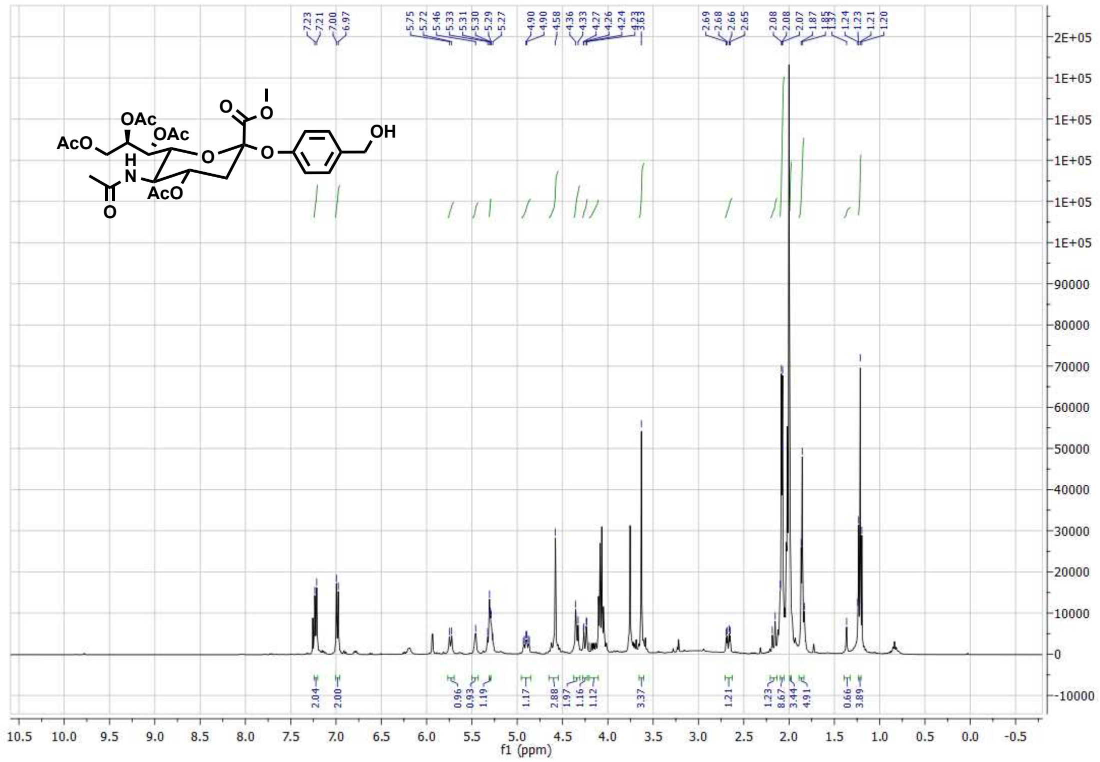

^{13}C-NMR spectra of compound **4**

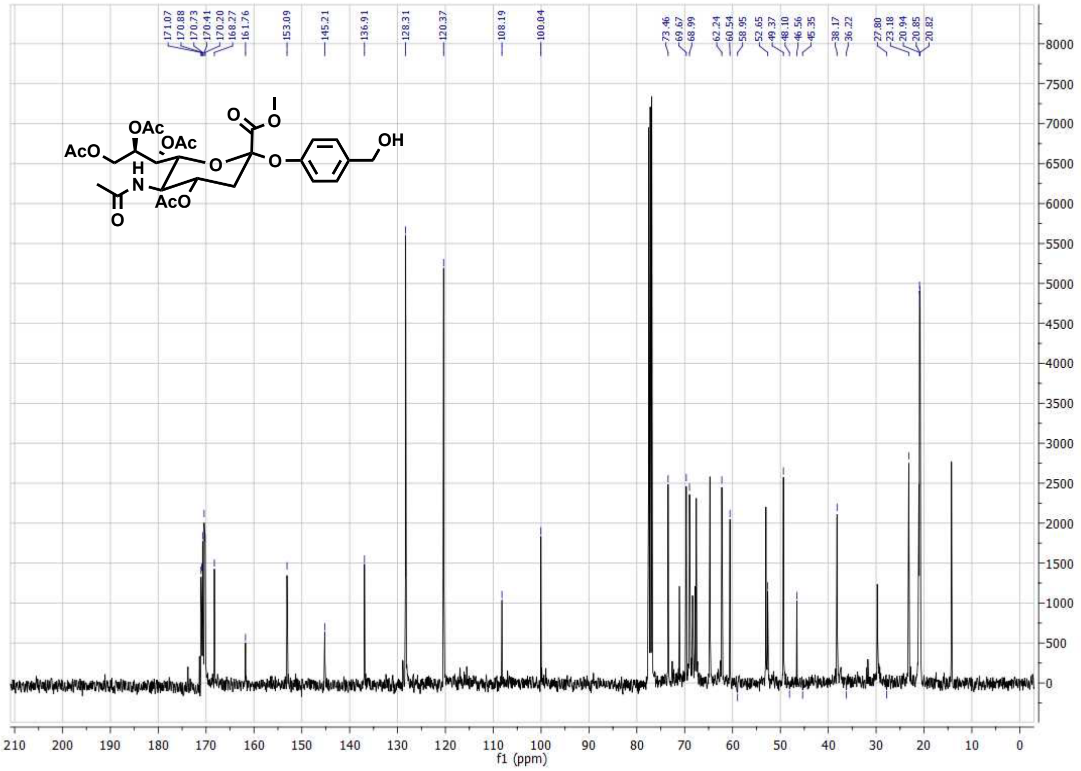

^{1}H-NMR spectra of compound **5**

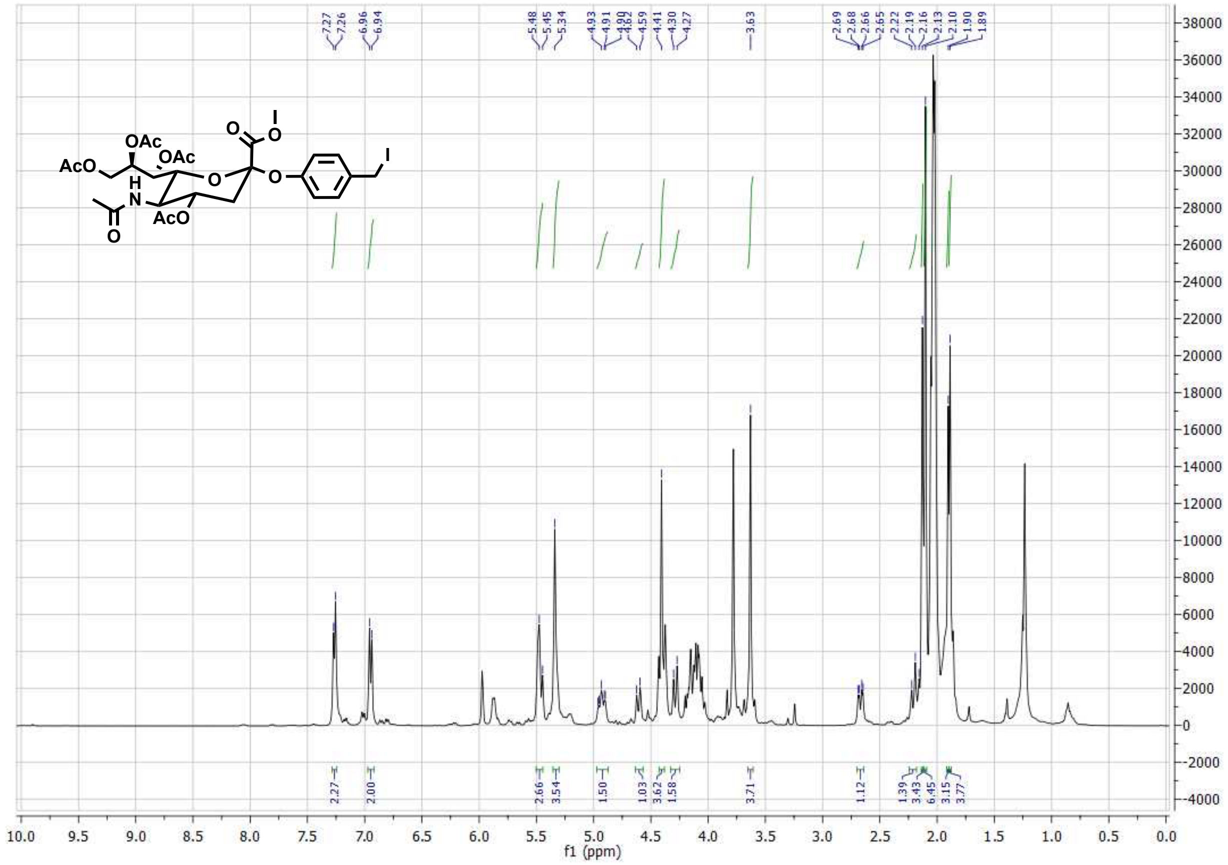

^{13}C-NMR spectra of compound **5**

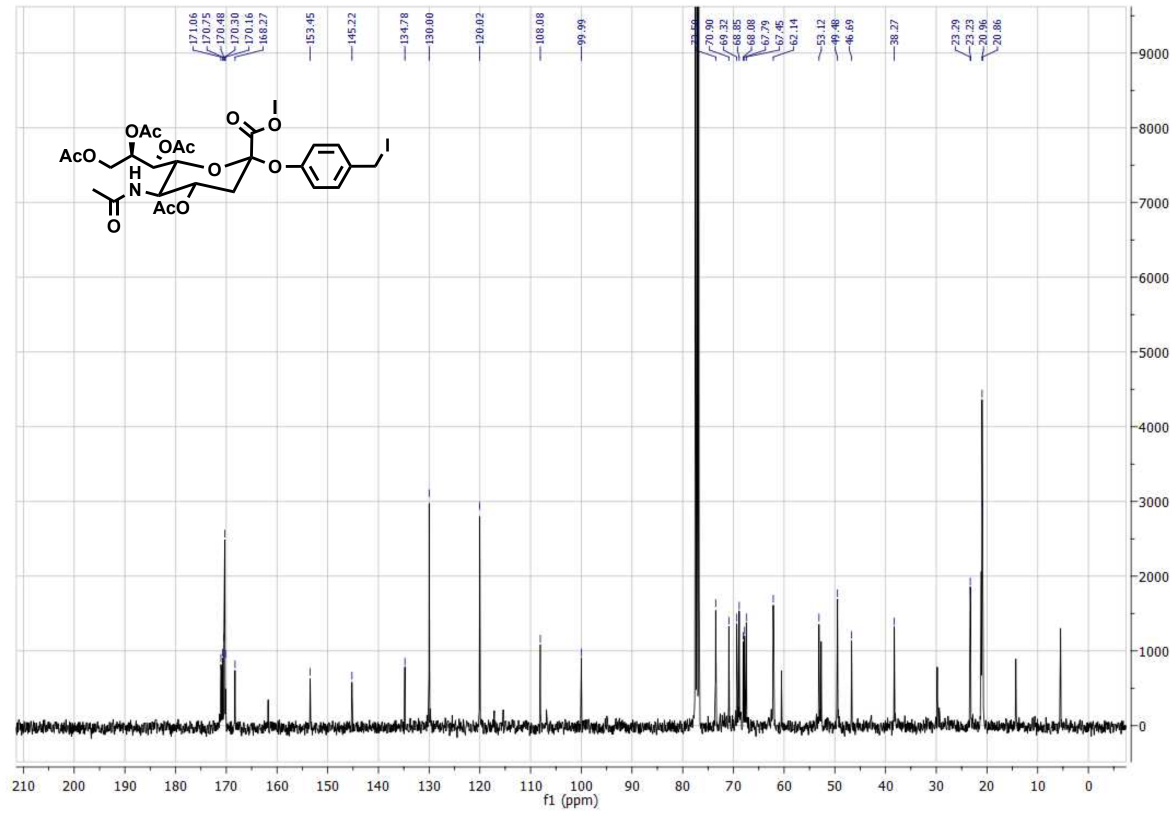

^{1}H-NMR spectra of compound **6**

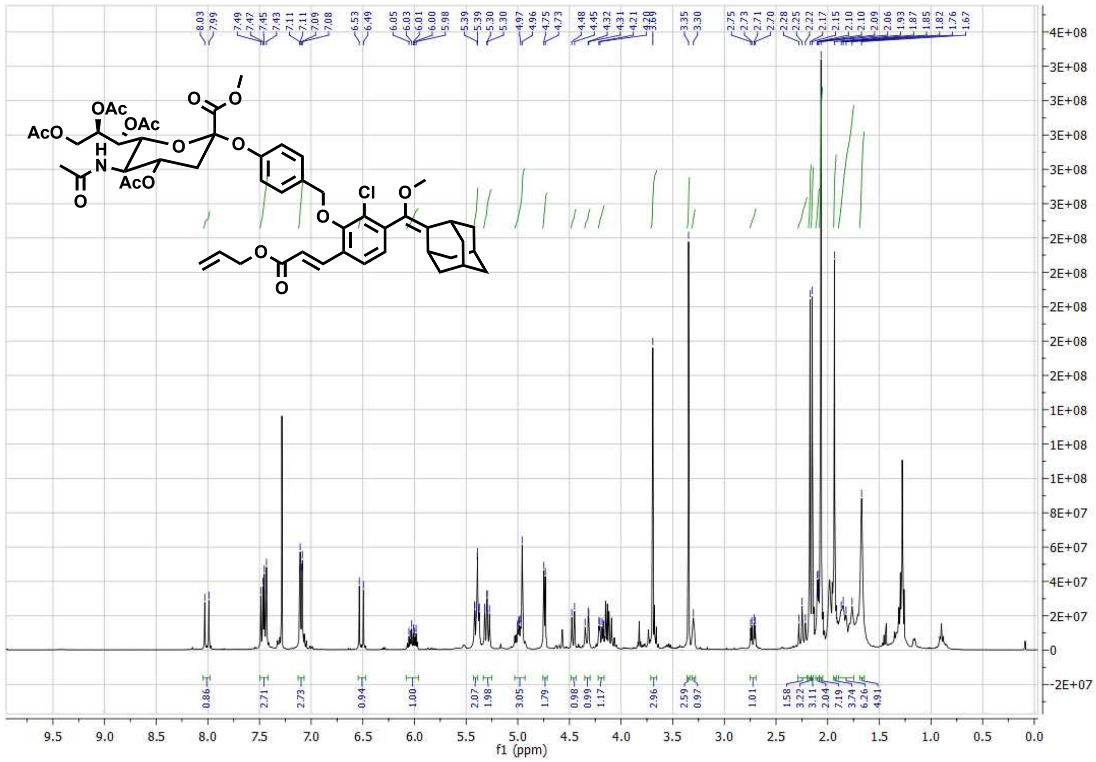

^{13}C-NMR spectra of compound **6**

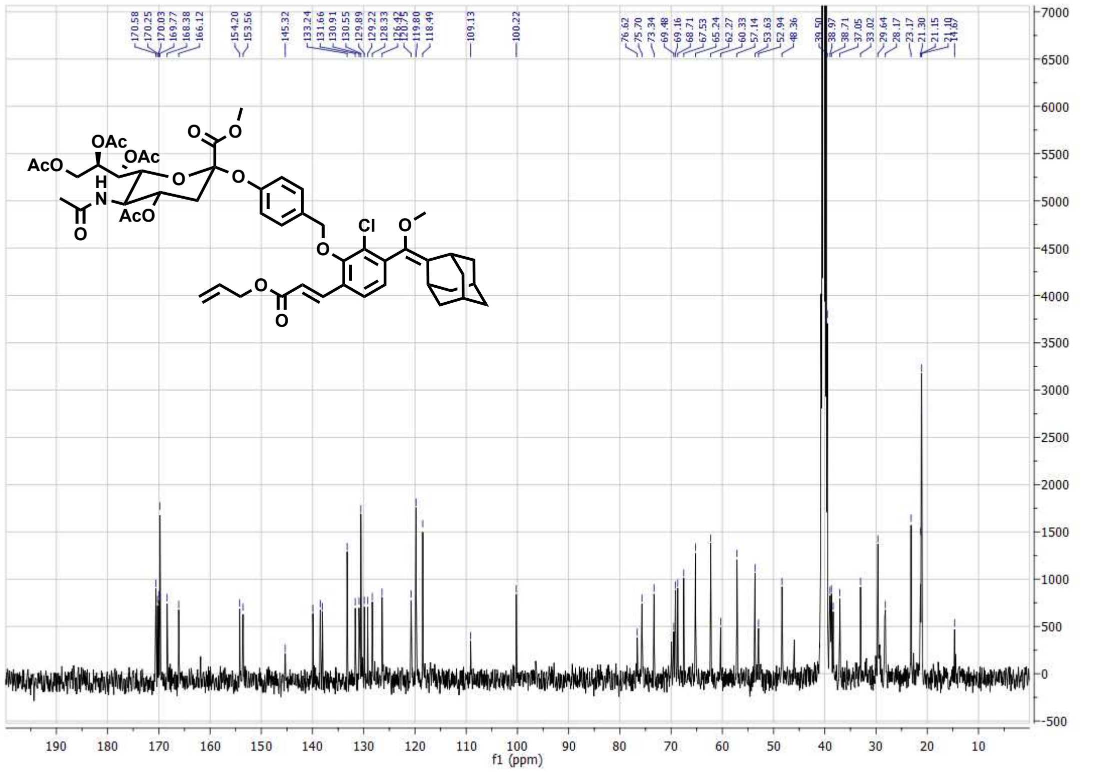

^{1}H-NMR spectra of compound **7**

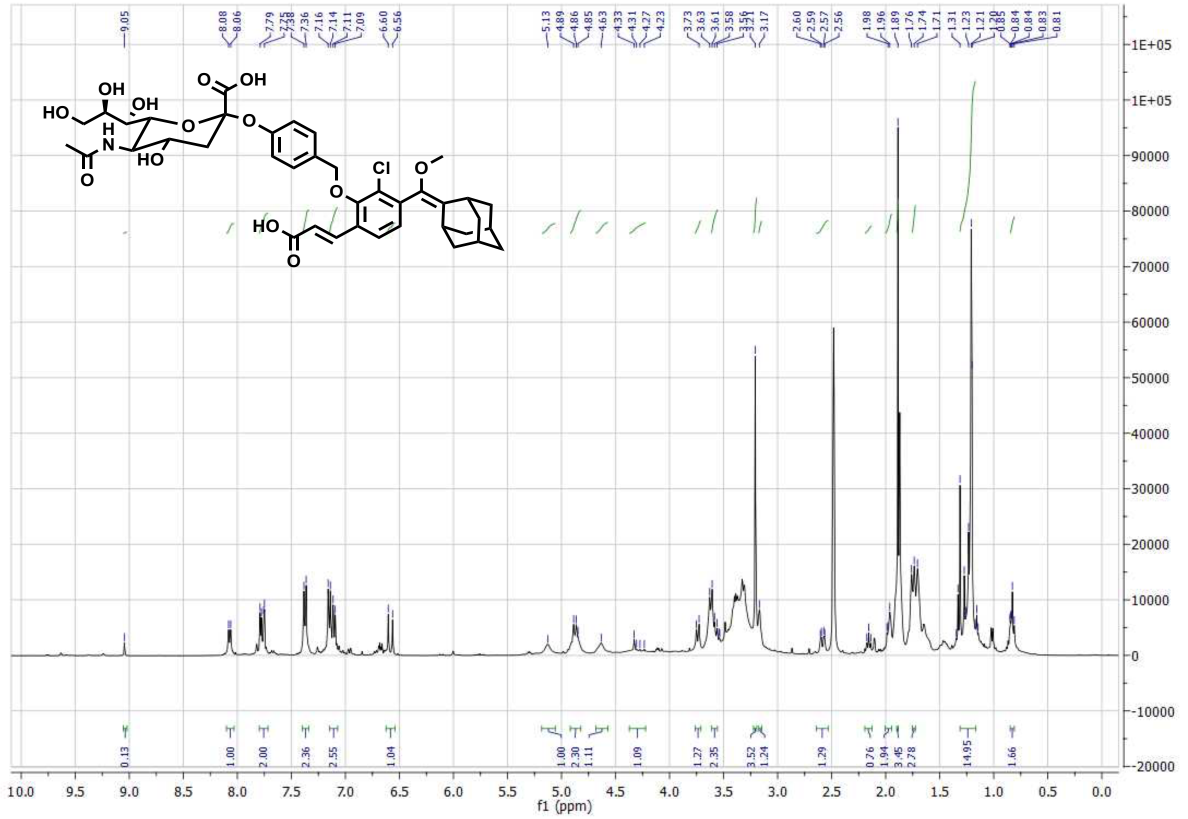

^{13}C-NMR spectra of compound **7**

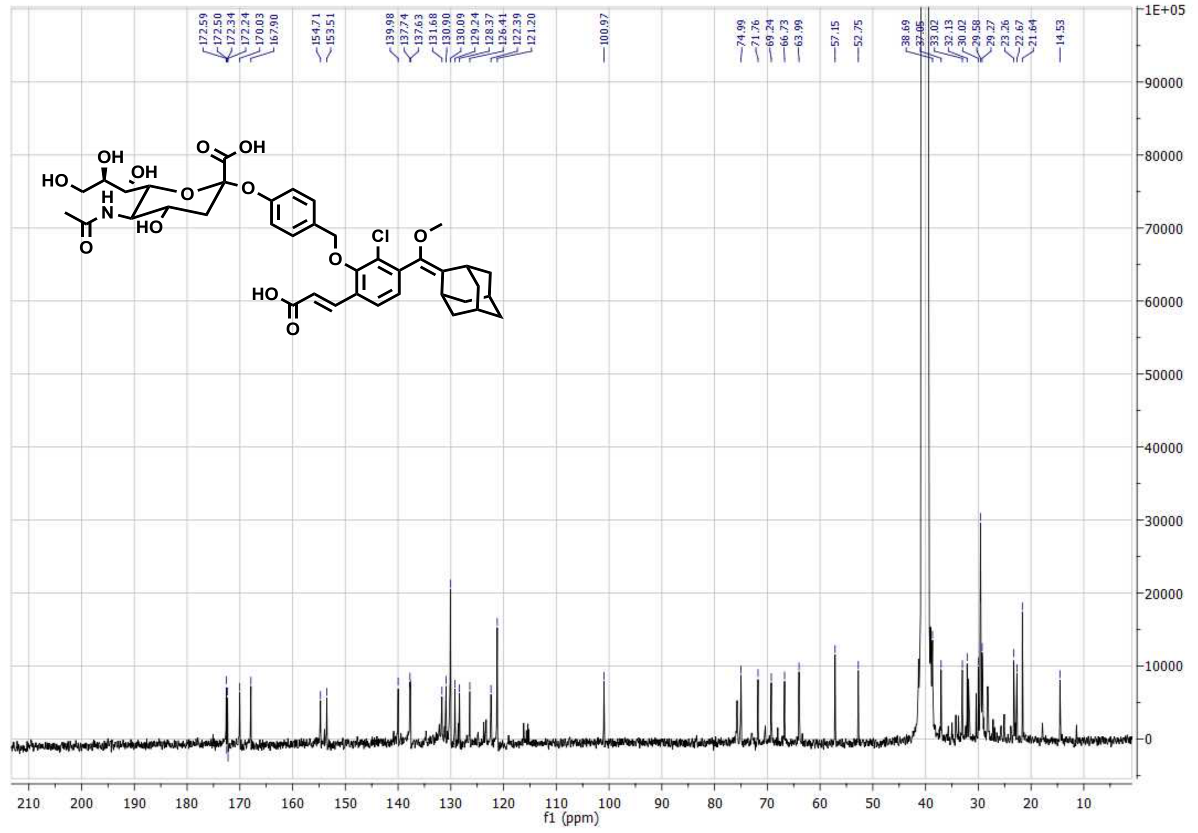

^{1}H-NMR spectra of compound **CLNA**

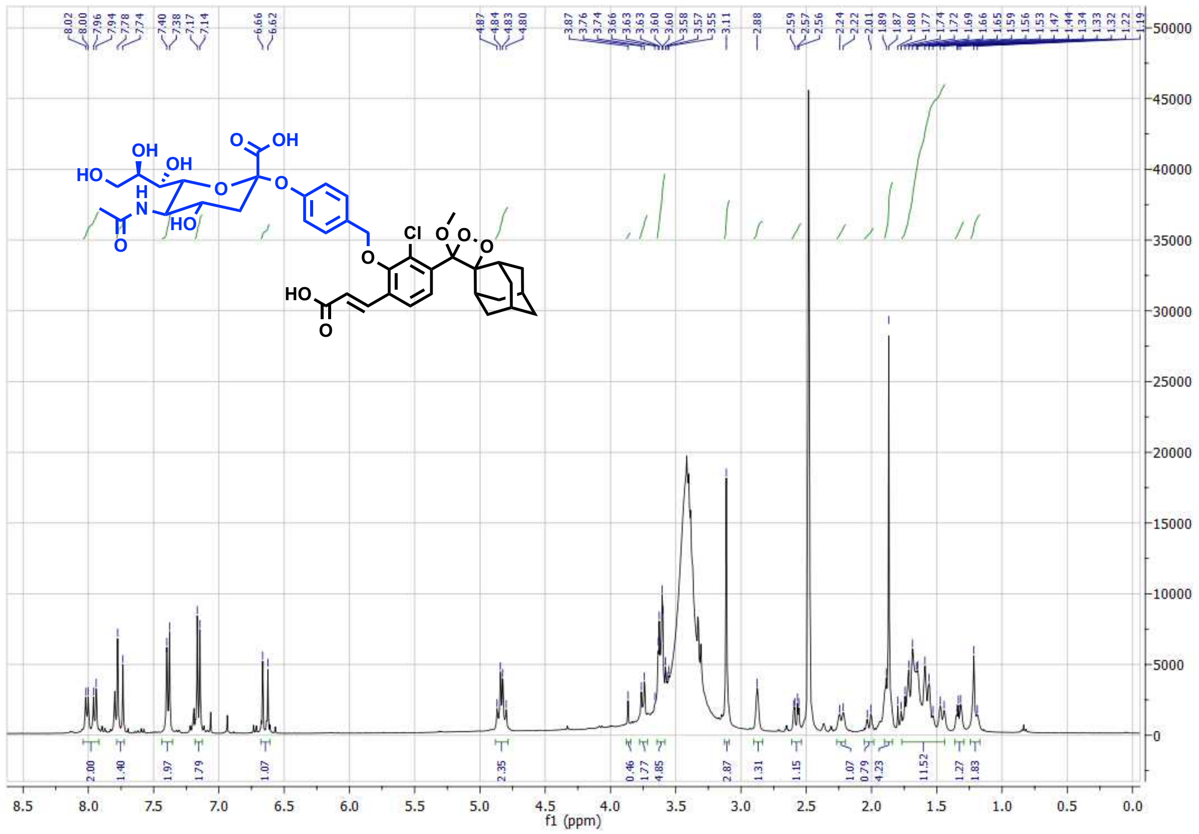

^{13}C-NMR spectra of compound **CLNA**

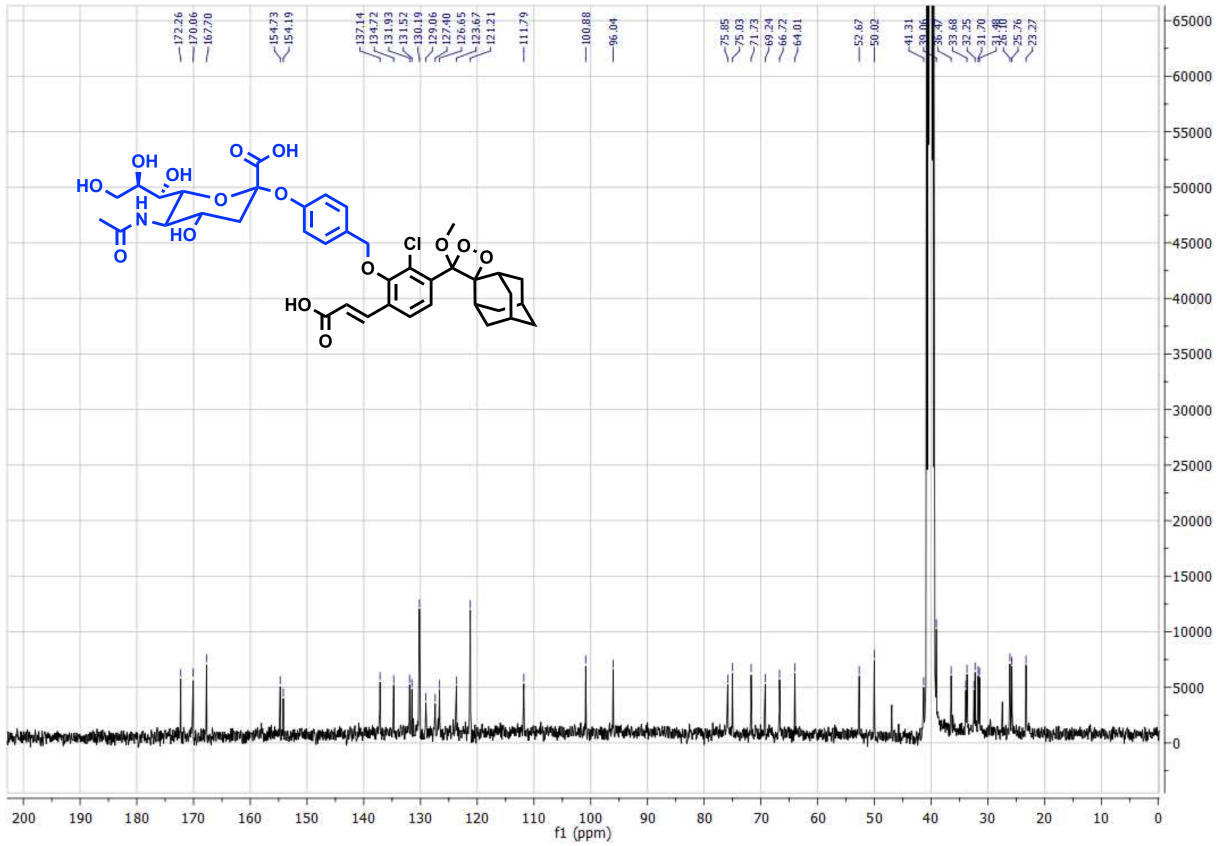

MS and/or HPLC Spectra of Key Compounds

3D HPLC spectra of **CLNA**

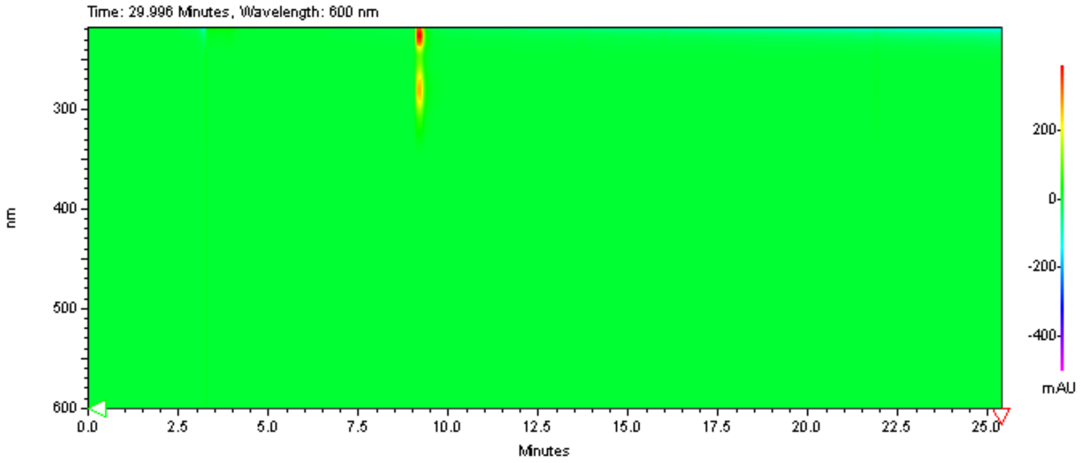

2D HPLC spectra of **CLNA**
*Absorbance measured at 280nm

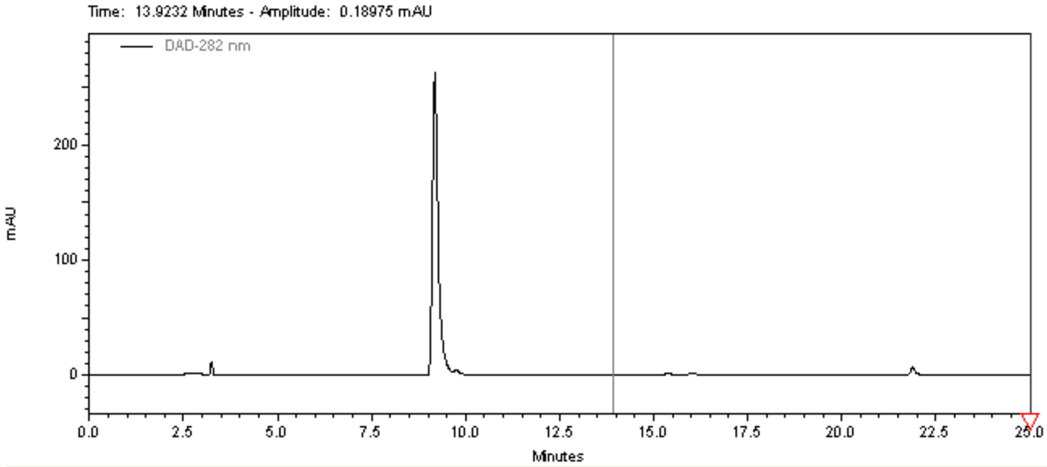

References

1 R. C. Buxton, B. Edwards, R. R. Juo, J. C. Voyta, M. Tisdale and R. C. Bethell, *Anal. Biochem.,* 2000, **280**, 291–300.

2 N. Hananya, J. P. Reid, O. Green, M. S. Sigman and D. Shabat, *Chem. Sci.*, 2019, **10**, 1380–1385.

3 FH. Niesen, H. Berglund, M. Vedadi, *Nat. Protoc.,* 2007, **2** *(9),* 2212–2221.
